# Evaluation of shared genetic aetiology between osteoarthritis and bone mineral density identifies *SMAD3* as a novel osteoarthritis risk locus

**DOI:** 10.1093/hmg/ddx285

**Published:** 2017-07-19

**Authors:** Sophie Hackinger, Katerina Trajanoska, Unnur Styrkarsdottir, Eleni Zengini, Julia Steinberg, Graham R.S. Ritchie, Konstantinos Hatzikotoulas, Arthur Gilly, Evangelos Evangelou, John P. Kemp, arcOGEN Consortium, GEFOS Consortium, David Evans, Thorvaldur Ingvarsson, Helgi Jonsson, Unnur Thorsteinsdottir, Kari Stefansson, Andrew W. McCaskie, Roger A. Brooks, Jeremy M. Wilkinson, Fernando Rivadeneira, Eleftheria Zeggini

**Affiliations:** 1 Human Genetics, Wellcome Trust Sanger Institute, Hinxton CB10 1HH, UK; 2 Departments of Internal Medicine and Epidemiology, Erasmus University Medical Center, Rotterdam 3000 CA, The Netherlands; 3 deCODE Genetics, Sturlugata 8, IS-101 Reykjavik, Iceland; 4 Department of Oncology and Metabolism, University of Sheffield, Sheffield S10 2RX, UK; 5 5th Department, Dromokaiteio Psychiatric Hospital, Athens 124 61, Greece; 6 Department of Hygiene and Epidemiology, University of Ioannina Medical School, Ioannina 45110, Greece; 7 Department of Epidemiology and Biostatistics, School of Public Health, Imperial College London, London, UK; 8 MRC Integrative Epidemiology Unit, University of Bristol, Bristol, UK; 9 University of Queensland Diamantina Institute, Translational Research Institute, Brisbane, QLD, Australia; 10 Department of Orthopedic Surgery, Akureyri Hospital, 600 Akureyri, Iceland; 11 Faculty of Medicine, University of Iceland, 101 Reykjavik, Iceland; 12 Institution of Health Science, University of Akureyri, 600 Akureyri, Iceland; 13 Department of Medicine, Landspitali, The National University Hospital of Iceland, 101 Reykjavik, Iceland; 14 Division of Trauma & Orthopaedic Surgery, University of Cambridge, Box 180, Addenbrooke’s Hospital, Cambridge CB2 0QQ, UK

## Abstract

Osteoarthritis (OA) is a common complex disease with high public health burden and no curative therapy. High bone mineral density (BMD) is associated with an increased risk of developing OA, suggesting a shared underlying biology. Here, we performed the first systematic overlap analysis of OA and BMD on a genome wide scale. We used summary statistics from the GEFOS consortium for lumbar spine (*n =* 31,800) and femoral neck (*n =* 32,961) BMD, and from the arcOGEN consortium for three OA phenotypes (hip, *n*_cases_=3,498; knee, *n*_cases_=3,266; hip and/or knee, *n*_cases_=7,410; *n*_controls_=11,009). Performing LD score regression we found a significant genetic correlation between the combined OA phenotype (hip and/or knee) and lumbar spine BMD (*r*_g_=0.18, *P =* 2.23 × 10^−2^), which may be driven by the presence of spinal osteophytes. We identified 143 variants with evidence for cross-phenotype association which we took forward for replication in independent large-scale OA datasets, and subsequent meta-analysis with arcOGEN for a total sample size of up to 23,425 cases and 236,814 controls. We found robustly replicating evidence for association with OA at rs12901071 (OR 1.08 95% CI 1.05–1.11, *P*_meta_=3.12 × 10^−10^), an intronic variant in the *SMAD3* gene, which is known to play a role in bone remodeling and cartilage maintenance. We were able to confirm expression of *SMAD3* in intact and degraded cartilage of the knee and hip. Our findings provide the first systematic evaluation of pleiotropy between OA and BMD, highlight genes with biological relevance to both traits, and establish a robust new OA genetic risk locus at *SMAD3*.

## Introduction

Osteoarthritis (OA) is a degenerative disease of the joints affecting over 40% of people over 70 years (1). Hallmarks of OA include cartilage degradation, joint-space narrowing, formation of osteophytes within the joint and subchondral bone remodeling (2,3). Due to a lack of therapeutic options, the main treatment strategy consists of pain management and, in severe cases, joint replacement surgery (2). OA is a complex disorder with the 18 currently known risk loci accounting for approximately 11% of disease heritability (4). The biggest OA genome-wide association study (GWAS) published to date was conducted by the arcOGEN consortium in a two-stage design, culminating in a total discovery sample of 7,410 cases and 11,009 controls (5,6). Cases in the arcOGEN study had radiographic hip or knee OA (defined as a Kellgren Lawrence score ≥ 2), and approximately 80% had undergone total joint replacement surgery, indicating disease progression to a severe degree (6).

In addition to establishing bigger OA sample collections, studying OA in the context of related phenotypes may add to the currently short list of established loci. While most GWAS to date have investigated a single phenotype, it is estimated that around 5% of risk variants and 16% of genes identified through these studies have pleiotropic effects on multiple phenotypes (7). In recent years, the accumulation of large-scale genomic datasets has made it possible to study genetic pleiotropy by incorporating data from multiple epidemiologically linked traits. Leveraging available summary data of correlated traits to identify common genetic risk factors can help pinpoint causal pathways and refine our understanding of disease mechanisms. OA in itself is a heterogeneous disorder, with heritability varying depending on the affected joint. Of the 18 published risk loci to date, 6 and 7 are associated with knee OA only and hip OA only, respectively, while 5 are associated with both hip and knee OA (4). This further highlights how phenotypic variation is reflected by genetics, and demonstrates the need for strict phenotype definitions.

The link between bone mineral density (BMD) and OA was first reported in 1972 by Foss and Byers, who observed higher BMD in femoral heads excised during OA-related hip replacement surgery (8). Since then, a number of cross-sectional and longitudinal studies have found higher femoral neck (FN) and lumbar spine (LS) BMD, as well as total body BMD to be associated with incident OA at the hip, knee and other joint sites (9–12).

Findings with regards to the relationship between BMD and OA progression are less clear (13). Elevated bone turnover – usually a marker for decreased BMD – was reported in patients with progressive knee OA compared to patients with stable OA (14). Decreased baseline femoral neck BMD (FNBMD) has also been associated with knee OA progression (15,16). Conversely, data from the Rotterdam study showed a non-significant trend of higher odds of knee OA progression with increased lumbar spine BMD (LSBMD) (17), while another study found no link between knee OA progression and total body- or FNBMD (9).

The largest genetic studies on BMD to date have been carried out by the Genetic Factors for Osteoporosis (GEFOS) consortium: a large-scale GWAS in 2012 found 56 loci associated with BMD (18); more recently, a rare variant of large effect was identified combining whole-genome sequencing and GWAS imputation (19). Several biological mechanisms are implicated in both OA and BMD, such as bone remodeling, mesenchymal stem cell differentiation and inflammation (2,6,18,20). *RUNX2*, a key transcription factor regulating endochondral ossification and osteoblast differentiation (21,22), has been associated with both OA and BMD based on its proximity to genome-wide significant variants (6,18). The other locus with known GWAS hits for both traits is *KLHL42* (or *KLHDC5*), although its biological relevance remains unclear (6,18).

In addition, Yerges-Armstrong and colleagues have previously shown nominal association of four BMD-linked single nucleotide polymorphisms (SNPs) with knee OA (23). However, despite the long-established epidemiologic link and shared biology, the genetic overlap of OA and BMD has not yet been assessed on a genome-wide level. Here, we present results from the first genome-wide analysis establishing shared genetic aetiology between OA and BMD.

## Results

### Known OA risk loci in BMD

Six loci associated with OA were nominally significant for FN- or LSBMD, including the *SUPTH3/CDC5L* locus, which is close to *RUNX2*, and *KLHL42/PTHLH* (Supplementary Material, Table S1).

### Genome-wide correlation

**Figure 1.**
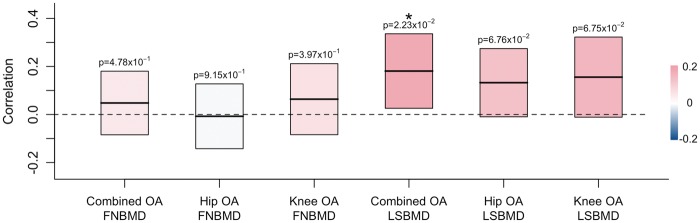
Genetic correlation between osteoarthritis (OA) and bone mineral density (BMD) as estimated by LD score regression. Rectangles show the correlation estimate (middle horizontal line) and standard errors (upper and lower bounds) of each comparison. Rectangles are coloured according to the strength of correlation. Significant correlation estimates are marked by an asterisk. LSBMD = lumbar spine BMD; FNBMD = femoral neck BMD.

We used linkage disequilibrium (LD) score regression to get an estimate of the genome-wide genetic correlation between OA and BMD. There was a significant correlation between combined OA and LSBMD (correlation = 0.18; *P =* 0.022), but not hip or knee OA and LSBMD or between FNBMD and any OA phenotype (Fig. 1).

### Extent of shared association signals

We found evidence for significant overlap of association signals at different *P*-value thresholds (*P*_t_) between all three OA categories and LSBMD (permutation adjusted *P*-value (*P*_perm_)<0.05) (Table 1).

**Table 1.** SNP-based overlap analysis of OA and BMD. For each comparison, the total number of SNPs present in both datasets after LD-pruning is given, as well as the number of SNPs falling below each *P*-value threshold (*P*_t_). *P*_perm_ = empirical overlap p-value obtained through permutation analysis

	Combined OA				Hip OA				Knee OA			
	LSBMD		FNBMD		LSBMD		FNBMD		LSBMD		FNBMD	
Total number of SNPs	75,015		75,161		74,999		75,147		75,125		75,270	
*P*_t_	SNPs	P_perm	SNPs	P_perm	SNPs	P_perm	SNPs	P_perm	SNPs	P_perm	SNPs	P_perm
0.5	28,940	8.53 × 10^−2^	27,599	2.74 × 10^−1^	28,563	1.64 × 10^−1^	27065	7.33 × 10^−1^	28,910	7.95 × 10^−1^	27,534	5.84 × 10^−2^
0.1	2,633	7.92 × 10^−2^	2,119	5.41 × 10^−1^	2,513	1.46 × 10^−2^	2057	1.74 × 10^−1^	2,620	3.67 × 10^−2^	2,131	2.33 × 10^−1^
0.05	931	1.25 × 10^−2^	698	2.35 × 10^−1^	868	1.42 × 10^−2^	647	2.47 × 10^−1^	939	8.98 × 10^−4^	700	3.10 × 10^−2^
0.04	682	2.41 × 10^−3^	486	2.33 × 10^−1^	641	6.48 × 10^−4^	434	4.99 × 10^−1^	680	4.74 × 10^−4^	474	1.27 × 10^−1^
0.03	451	1.20 × 10^−3^	314	6.57 × 10^−2^	416	1.02 × 10^−3^	272	4.20 × 10^−1^	437	2.87 × 10^−3^	291	3.27 × 10^−1^
0.02	251	1.95 × 10^−3^	160	1.07 × 10^−1^	239	6.10 × 10^−5^	150	6.31 × 10^−2^	244	1.59 × 10^−3^	152	3.03 × 10^−1^
0.01	87	2.79 × 10^−2^	60	5.84 × 10^−2^	87	2.97 × 10^−4^	52	4.94 × 10^−2^	88	3.01 × 10^−3^	53	2.18 × 10^−1^
0.005	37	6.89 × 10^−3^	24	2.43 × 10^−2^	38	3.04 × 10^−4^	26	6.39 × 10^−4^	29	1.97 × 10^−1^	16	6.94 × 10^−1^
0.001	10	3.90 × 10^−5^	3	4.52 × 10^−2^	7	1.68 × 10^−3^	1	1.00	6	1.12 × 10^−2^	4	3.39 × 10^−2^
5 × 10^−4^	4	6.81 × 10^−3^	0	1.00	6	5.70 × 10^−5^	1	5.64 × 10^−2^	4	4.21 × 10^−3^	2	7.12 × 10^−2^

Analysis of the combined OA and LSBMD data resulted in significant overlap *P*-values at *P*_t_=0.001 and 0.005, as well as at less stringent *P*_t_. Four SNPs overlap at *P*_t_=5×10^−4^ (rs17158899, rs4536164, rs11826287 and rs630765), one of which (rs11826287) is genome-wide significantly associated with FNBMD (*P =* 3.61×10^−14^) and maps to an intron in *LRP5*.

The highest overlap was observed between hip OA and LSBMD, with six SNPs overlapping at *P*_t_=5×10^−4^ (*P*_perm_=5.7×10^−5^). Two of these SNPs are genome-wide significantly associated with BMD in GEFOS (rs1524928, *P =* 5.29×10^−9^ and rs716255, *P =* 2.07×10^−11^). A significant overlap was also observed for *P*_t_ of 0.001, 0.005 and 0.01 in the hip OA-LSBMD comparison.

Compared to the hip OA and LSBMD analysis, overlap *P*-values for the knee OA and LSBMD comparison were at least one order of magnitude smaller. Four SNPs (rs7104420, rs9466056, rs881803 and rs4536164) overlapped at *P*_t_=5×10^−4^ in this analysis (*P*_perm_ =4.21×10^−3^). Two of these, rs4536164 and rs9466056, fall within known BMD risk loci (18).

Overlap signal was much weaker for the OA and FNBMD comparisons, with only five *P*_t_ reaching statistical significance (*P*_t_ = 0.001 for knee OA, *P*_t_ = 0.005 and 0.01 for hip OA, and *P*_t_ = 0.001 and *P*_t_ = 0.005 for combined OA). The SNP overlapping at *P*_t_=5×10^−4^ for hip OA and FNBMD (rs1524928) was also among the six SNPs identified in the hip OA and LSBMD analysis. Two SNPs overlapped at this *P*_t_ for knee OA, one being rs9466056 and the other rs1283614, which maps to an intron of the BMD locus *MEF2C* (24).

### Evidence for colocalising regions

We employed a regional Bayesian colocalisation test that measures the posterior probabilities (PP) for each of four alternative hypotheses compared to one global null hypothesis (*i.e.* no associations in that region). We identified four independent genomic regions with a high posterior probability (PP) of harbouring one causal variant common to both traits analysed (PP for hypothesis 3 ≥ 0.9) (Table 2). The region containing the *RPS6KA5* gene was identified by three comparisons (combined OA and LSBMD, hip OA and LSBMD, and hip OA and FNBMD). The most strongly associated SNPs in this region have genome-wide significant associations with increased BMD at both the LS and FN (rs1286147 and rs1286063, *P < *5×10^−8^) and nominally significant associations with increased risk of combined and hip OA (rs1286077, *P < *0.05); the three SNPs are in perfect LD (*r*^2^=1.00 for each pairwise combination).

**Table 2.** Regions with strong evidence of pleiotropy. For each region the number of SNPs, start and stop position in basepairs (bp) and most strongly associated SNPs for OA and BMD are given. Chromosome coordinates are in hg19. Hypothesis 3 = one causal variant; hypothesis 4 = two distinct causal variants; PP = posterior probability

	Analysis	SNPs	Chr	Start (bp)	Stop (bp)	Top SNP BMD	Top SNP OA	PP
**Hypothesis 3**	**combinedOA and LSBMD**	817	chr14	91297823	93129850	rs1286147; rs1286063	rs1286077	0.95
	**hipOA and FNBMD**	817	chr14	91297823	93129850	rs1286147	rs1286077	0.98
	**hipOA and LSBMD**	817	chr14	91297823	93129850	rs1286147; rs1286063	rs1286077	0.99
	**hipOA and LSBMD**	1242	chr10	78708452	80875213	rs7071206	rs716255	0.92
	**hipOA and LSBMD**	531	chr1	44974119	46897698	rs7554123	rs7545984	0.91
	**kneeOA and FNBMD**	1235	chr6	19208477	21677746	rs9466056	rs9466056	0.99
**Hypothesis 4**	**hipOA and LSBMD**	268	chr4	696848	1415698	rs3755955	rs3755920	0.97
	**kneeOA and LSBMD**	382	chr16	14464538	16152940	rs4985155	rs9935327	0.95
	**kneeOA and LSBMD**	1070	chr6	150255029	151910904	rs4869742	rs9384514	0.90

Two further regions were identified in the hip OA and LSBMD analysis; one of these spans a known BMD locus close to the *KCNMA1* gene, while the other does not contain genome-wide significant variants for either OA or BMD.

The region identified in the knee OA and FNBMD analysis contains one lead SNP for both traits, rs9466056, which is a known variant associated with high FN and LSBMD mapping to an intergenic region between *CDKAL1* and *SOX4*.

We also identified three regions (Table 2) with a high PP of harbouring two distinct causal variants (PP for hypothesis 4 ≥ 0.9). All three of these contain a known BMD locus, with the top SNPs for LSBMD mapping to introns of *IDUA*, *CCDC170* and *PDXDC1*. The top SNPs for knee and hip OA are nominally associated (*P < *0.05) with these respective phenotypes in arcOGEN.

### Gene and pathway analysis

Of the individual genes significantly associated (*Q* < 0.05) with at least one OA or BMD phenotype, *SUPTH3, COL11A1*, and *APCDD1* overlapped between OA and BMD (Supplementary Material, Table S2). All three include variants that were identified in the SNP-wise overlap analysis and taken forward for replication.

There were no pathways significantly associated with any OA phenotype in any of the analyses. One of the CP pathways was associated with FNBMD (“basal cell carcinoma”, *Q =* 0.02) when allowing a 20 kilobase (kb) window around genes. Using GO annotations, a total of 33 unique pathways were associated with either BMD phenotype using strict or lenient gene definitions (Supplementary Material, Tables S3–S5), including several with direct biological relevance, such as “regulation of ossification” or “osteoblast development”.

### Cross-phenotype meta-analysis

To search for potential novel associations not identified by single-trait GWAS, we performed a cross-phenotype meta-analysis between each pairwise combination of OA and BMD datasets. Using the CPASSOC method (25), we computed two statistics, S_hom_ and S_het_, which assume homogeneous and heterogeneous effects across studies, respectively. The quantile–quantile and Manhattan plots of these analyses are shown in Supplementary Material, Figures S1–S4. We identified 13 independent associations not previously reported for BMD or OA, which we followed up in the UK Biobank combined OA dataset (Supplementary Material, Table S10). One SNP, rs11164649, was nominally significant (*P < *0.05). This SNP lies in an intron of the *COL11A1* gene and is in strong LD (*r*^2^=0.92) with a variant (rs1903787) identified in the SNP-wise overlap analysis which was taken forward for replication.

### Replication and meta-analysis for OA

We meta-analysed 143 SNPs identified in the colocalisation and/or *P*-value based overlap analysis in arcOGEN, UK Biobank and deCODE (Supplementary Material, Table S6). None of the SNPs taken forward are genome-wide significantly associated with arcOGEN. We found a significant excess of independent SNPs with the same direction of effect in all three meta-analysis cohorts among variants with *P*_meta_<0.05 (binomial sign test *P =* 7.75×10^−11^), as well as all independent SNPs included in the meta-analysis (binomial sign test *P =* 0.03).

Variants within several genes linked to bone, cartilage and extracellular matrix biology, including *APCDD1, SUPTH3, COL11A1, NOTCH4, SEMA3A, LGR4, PTCH1* and *RPS6KA5*, were associated at *P*_meta_<0.05 (Supplementary Material, Table S6).

Two variants reached genome-wide significance in the meta-analysis across arcOGEN, deCODE and UK Biobank: rs12901071 (OR 1.08 95% CI 1.05–1.11, *P*_meta_=3.12×10^−10^) and rs10518707 (OR 1.07, 96% CI 1.03–1.09, *P*_meta_=2.15×10^−8^). Both are intronic variants in the *SMAD3* gene (*r*^2^=0.645) and were identified in the SNP-wise overlap analysis of combined OA vs. LSBMD and hip OA vs. LSBMD, respectively.

**Figure 2.**
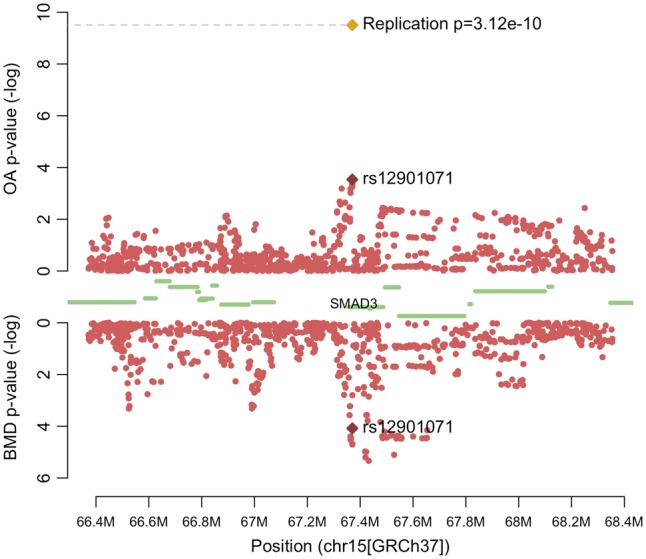
Regional association plot of *SMAD3.* The -log(p-values) of SNPs in the arcOGEN combined osteoarthritis (OA) data (top) and GEFOS lumbar spine bone mineral density (LSBMD) data (bottom) are plotted against their chromosomal position. The meta-analysis p-value of rs12901071 is plotted as a golden diamond. Protein coding genes are represented by green bars.

Both new genome-wide significant SNPs for OA were imputed in the arcOGEN data (imputation info score > 0.95) and are nominally associated with combined OA (Supplementary Material, Table S6; Fig. S2). They are also nominally associated with increased LSBMD in GEFOS (rs12901071, *P =* 1.58×10^−3^ and rs10518707, *P =* 3.47×10^−5^) (Fig. 2), but not FNBMD (rs12901071, *P =* 3.72×10^−1^ and rs10518707, *P =* 2.46×10^−1^). *SMAD3* is associated (*P < *0.05) with LS and FNBMD, hip and combined OA in the gene analysis (Supplementary Material, Table S7), although this association only holds for LSBMD after using false discovery rate (FDR) correction (*Q =* 6.92×10^−6^).

In the colocalisation analysis, the region in which both top SNPs reside (chr15:67,095,629-69,017,421) has a PP of containing a single pleiotropic variant associated with hip OA and LSBMD (hypothesis 3) of 0.88.

### Functional follow-up of *SMAD3*

Using RNA sequencing data, we confirmed the expression of *SMAD3* in low-grade degenerate articular cartilage of 12 knee and 9 hip OA patients undergoing total joint replacement (26) (Supplementary Material, Fig. S5). *SMAD3* is among the 30% most expressed genes in the knee articular cartilage samples, and among the 15% most expressed genes in the hip articular cartilage samples.

## Discussion

The analysis of shared genetic aetiology across epidemiologically linked traits can enhance power to identify disease variants and shed light into the biological mechanisms underpinning these associations. We conducted the first genome-wide overlap analysis of BMD and OA using summary statistics from the two largest GWAS of these two traits.

We identified 143 variants with evidence for association with OA and BMD. Many of these reside in or near biologically relevant genes, two of which (*KLHL42*/*KLHDC5* and *SUPT3H/RUNX2*) are established loci for both traits (6,18). Variants in three loci (*SUPTH3*, *APCDD1*, and *COL11A1*) were also significantly associated with at least one OA and BMD phenotype in the gene analysis. *APCDD1* is an inhibitor of *WNT* signaling (27), which is implicated in both OA and BMD. *COL11A1* encodes collagen type 11, an important component of cartilage and bone, and has been associated with OA in a candidate gene meta-analysis (28). Other examples include the *LGR4* gene, in which a rare variant in the Icelandic population has been associated with low BMD and osteoporotic fractures (29); and *SEMA3A*, which affects bone remodeling in rats (30).

We identified novel genome-wide significant associations at two intronic SNPs in *SMAD3*, and confirm expression of this gene in primary chondrocytes from articular cartilage of OA patients undergoing total joint replacement surgery. Activated SMAD3 acts downstream of TGF-β, repressing osteoblast differentiation and the production of bone matrix (31,32). It also represses the cartilage-degrading enzyme matrix metalloproteinase 13 in chondrocytes (32). Missense mutations in a conserved protein domain of *SMAD3* have been linked to aneurysm-osteoarthritis syndrome, a congenital disorder characterised by arterial aneurysms, heart abnormalities and early-onset OA (33).

Due to its role in bone and cartilage biology, *SMAD3* has been previously assessed in a candidate gene study of hip and knee OA (34). Despite their small sample size (number of cases < 400), the investigators found nominal associations (*P < *0.05) for both OA phenotypes in their discovery, which were further strengthened in a meta-analysis (hip OA *P =* 4×10^−4^; knee OA *P =* 7.5×10^−6^). Notably, their top signal (rs12901499) maps to the same locus as our lead SNP (*r*^2^=0.645).

More recently, two studies have shown *SMAD3* expression to be correlated with the genotype at a 3’UTR SNP (35), and to be significantly higher in cartilage from OA patients compared to healthy controls (36). The authors postulate that this could be a compensatory mechanism to counteract existing cartilage damage, or that *SMAD3* expression levels outside a narrow range have detrimental effects.

We observed a stronger overlap between LSBMD and OA. The fact that only the correlation between combined OA and LSBMD was significant could be due to the bigger sample size in this OA dataset compared to the hip or knee OA data. While the FN- and LSBMD datasets are very similar in size, the knee OA and hip OA datasets each contain approximately half the number of cases compared to the combined OA dataset. This difference in power might at least partly explain why the genetic correlation estimates for joint-specific OA and LSBMD did not achieve statistical significance. Epidemiological data from the Chingford study have shown increased baseline BMD to be associated with incident radiographic knee OA, with the mean increase in LSBMD being approximately twice as high as the increase in FNBMD (10,15). Incident knee OA was also linked to higher baseline LSBMD, but not FNBMD, in the Baltimore Longitudinal Study of Ageing (37). The reasons for this differential association of FN- and LSBMD with OA remain unclear. One possible explanation could be the comorbidity of knee and spinal OA, characterised by spinal osteophytes, which could lead to increased LSBMD measurements. However, in one study, adjustment for the presence of osteophytes at the lumbar spine did not change the strength of association between OA and LSBMD (10). Damage to the spine accumulates over time and can lead to changes such as breakdown of the invertebral discs, scoliosis and osteochondrosis. This process is also referred to as degenerative disc disease (DDD). Although the association between DDD and LSBMD remains inconclusive (38–41), it is known that the presence of degenerative features can increase LSBMD measurements obtained via dual X-ray absorptiometry (42). While this may have contributed to the observed association between LSBMD and OA, we found genetic correlations of a similar magnitude between OA and skull, as well as total body BMD measurements in a paediatric cohort (43) (Supplementary Material, Table S11; Supplementary Methods). As DDD and related features such as osteophytes are unlikely to be present in young individuals, these results suggest that the correlation between OA and LSBMD is not purely artefactual.

Our analyses showed a greater degree of overlap between hip OA and both BMD measurements than between knee OA and BMD. Hip OA is estimated to have a higher heritability than knee OA (44), with environmental risk factors such as physical activity and BMI more strongly associated with the latter (45).

The analyses outlined here present the first comprehensive evaluation of genetic overlap between BMD and radiographic OA. Our results lend further support to the hypothesis of common genetic factors underlying these two traits, and establish *SMAD3* as a genome-wide significant risk locus for OA with a potential pleiotropic effect on BMD.

Our work exemplifies the potential to uncover new disease risk loci by combining data of epidemiologically linked traits. Recent efforts in the development of statistical methods to detect pleiotropy, as well as the establishment of cross-disorder working groups (46) and of biobank collections (47), have made it possible to systematically assess pleiotropy in human disease genetics. Methods combining univariate summary statistics of different traits – such as the colocalisation analysis employed here (48) – often do not require a locus to be genome-wide significantly associated with any of the individual studies to detect a cross-phenotype association. Hence, they can increase power to identify associated variants or regions without the need to collect larger sample sizes (49).

There is a stark difference in sample size and, consequently, statistical power between the arcOGEN and GEFOS GWAS datasets. Larger datasets where phenotype information for both OA and BMD is available in the same individuals will be necessary to further disentangle the extent of shared genetics between them. Pinpointing the common biological pathways of these two complex traits will provide insight into the underlying mechanisms of OA, facilitating the identification of novel targets or drug repurposing opportunities for its treatment.

## Materials and Methods

### Datasets

All analyses outlined here were conducted using summary association statistics from the arcOGEN (6) and GEFOS consortia (18). The arcOGEN data comprised three OA phenotypes: knee OA, hip OA, and knee and/or hip OA (combined OA). A detailed description of the contributing studies and phenotype definitions can be found in (18). Briefly, OA case status was determined radiographically as a Kellgren-Lawrence grade score ≥ 2. Most cases included in arcOGEN had progressed to a severe disease endpoint, as evident from the fact that 80% had undergone total joint replacement surgery.

For the *P*-value-based overlap analysis as well as the gene and pathway analysis, we excluded samples from London-based cohorts (TwinsUK and Chingford Study) from the arcOGEN datasets to avoid overlap with GEFOS. After exclusion of 714 samples, we carried out genome-wide association analyses on the arcOGEN dataset for each of the three phenotype groups using the “–method score” option in SNPTEST v2.5 (50). The full arcOGEN dataset was used for all other analyses described.

The BMD data consisted of meta-analysis summary statistics for FN and LSBMD (18).

For replication, we used summary statistics from two OA GWAS: the UK Biobank (47) and the deCODE study. Details on case definitions in UK Biobank and deCODE can be found in the Supplementary Methods.

Sample sizes for each dataset are outlined in Supplementary Material, Table S8.

### Genome-wide correlation

We performed LD score regression analysis (51) on each pairwise combination between the BMD and OA datasets, using pre-computed LD scores based on the European sample of the 1000 Genomes Project (52). We accounted for sample overlap between each pair of datasets through tetrachoric correlation of the Z-scores of SNPs present in both studies.

### Assessment of shared association signals

For each pairwise combination between the two BMD and three OA phenotypes we assessed the extent of shared signals at different p-value cutoffs, following the approach described by Elliott and colleagues (53). Briefly, we filtered both datasets to a common set of SNPs on which p-value-informed LD pruning was performed. To this end, SNPs were sorted based on their association with OA, and, starting with the lowest p-value, any SNP in LD with the index SNP (*r*^2^>0.05) was removed. The next SNP was then considered, and so on.

The extent of shared association signals between OA and BMD was assessed by counting the number of overlapping variants above and below ten different p-value thresholds (*P*_t_: 0.5, 0.1, 0.05, 0.04, 0.03, 0.02, 0.01, 0.005, 0.001, 5 × 10^−5^). To test for significance of overlap, a chi-squared test on the resulting 2×2 contingency tables was performed at each *P*_t_.

Empirical overlap p-values were obtained by repeating the chi-squared test after randomly permuting the GEFOS p-values. This was done 1,000,000 times to obtain a null distribution of overlap p-values against which the original overlap p-value could be compared.

### Colocalisation analysis

We employed a Bayesian colocalisation method to search for genomic regions harbouring cross-phenotype associations between OA and BMD (48). Briefly, the algorithm uses Z-scores and standard errors from two association studies to generate posterior probabilities for each of five hypotheses:

H_0_: the region contains no variants associated with trait 1 or trait 2

H_1_: the region contains one variant associated with trait 1

H_2_: the region contains one variant associated with trait 2

H_3_: the region contains one variant associated with both trait 1 and trait 2

H_4_: the region contains one variant associated with trait 1 and a second variant associated with trait 2

Genomic regions were defined according to approximately independent LD-blocks (54). Analogous to LD score regression, we used the tetrachoric correlation of the Z-scores of SNPs present in datasets as an estimate of sample overlap.

### Gene and pathway analysis

Gene- and pathway analyses were performed on each OA and BMD dataset using MAGMA (55). First, SNPs are assigned to genes, which are tested for their association with the phenotype. Results from this step are then combined into pathway-based association statistics.

For the gene analysis, we grouped variants into genes using SNP locations from dbSNP version 135 and NCBI 37.3 gene definitions. We performed this step twice, once annotating SNPs to a gene only if they fell within the gene’s transcription start and stop site, and once including SNPs that fell within a 20 kilobase window of the gene.

We ran two separate pathway analyses, one using the Molecular Signatures Database canonical pathways collection (56), comprising 1,329 manually curated gene-sets from nine databases (Supplementary Material, Table S9), and one using 6,166 gene-sets from the Gene Ontology pathway database (57).

Significance was defined using a 5% FDR equivalent to a *Q*-value of 0.05 for both the gene and pathway analyses (58).

### Cross-phenotype meta-analysis

We used a multi-trait extension to meta-analysis to search for novel associations in each pairwise combination of arcOGEN and GEFOS datasets (25). The method, CPASSOC, requires only summary statistics and generates two test statistics: the first, S_hom_, assumes homogeneous effects across studies and is equivalent to performing an inverse variance weighted meta-analysis if no sample overlap between the studies exists. The second, S_het_, is more powerful if effects are heterogeneous between studies. Both statistics require the specification of a correlation matrix between the included studies. To calculate this, we followed the approach by Park *et al.* (59), using all independent SNPs (*r*^2^ < 0.2) present in both the OA and BMD dataset that were not associated with either trait (−1.96 > Z score < 1.96), and taking the Pearson’s correlation of their Z-scores (25).

We performed an *in-silico* lookup in the UK Biobank hip and/or knee OA data of top SNPs with *P < *5×10^−8^ in any of the CPASSOC analyses that did not fall into known OA or BMD loci.

### Replication and meta-analysis for OA

We took forward a total of 143 SNPs for *in silico* replication. This set comprises the two most strongly associated variants (one for each trait) in each region from the Bayesian colocalisation test, as well as all variants overlapping at *P*_t_=0.005 in the SNP-based overlap analyses. We used the METAL (60) software package to perform inverse variance weighted meta-analysis of these SNPs in relation to hip and/or knee OA using summary statistics from the arcOGEN combined OA dataset (including London samples), the UK Biobank (47) and the deCODE (61) study.

### Functional follow-up of *SMAD3*

Samples were obtained and processed as in Ref. 25. Briefly, articular cartilage was obtained from 12 patients undergoing total joint replacement for knee OA, and 9 patients for hip OA. Cartilage was graded using the OARSI cartilage classification system (62,63).

## Supplementary Material

Supplementary Material is available at *HMG* online.

## Supplementary Material

Supplementary Tables and Figures
