# Supplementary material for: Evaluation of shared genetic aetiology between osteoarthritis and bone mineral density identifies SMAD3 as a novel osteoarthritis risk locus
Source: Hum Mol Genet. 2017 Jul 19;26(19):3850–8. doi: 10.1093/hmg/ddx285 (PMC5886098; doi:10.1093/hmg/ddx285)
Supplement: Supplementary Tables and Figures [file hmg-2017-tf-01185_hackinger_supplementary_data_ddx285.docx]

**Evaluation of shared genetics between osteoarthritis and bone mineral density identifies *SMAD3* as a novel osteoarthritis risk locus**

**Supplementary Data**

Supplementary Methods 2

Supplementary Tables 4

Supplementary Figure 19

arcOGEN consortium membership 20

GEFOS consortium membership 21

**Supplementary Methods**

**UK Biobank dataset**

Hospital episode statistics were used to define case status for OA in the UKBB sample. Inclusion and exclusion criteria were based on the International Statistical Classification of Diseases and Related Health Problems (ICD). Cases were defined as having OA (hip and/or knee) ICD-9 or ICD-10 codes only, and no inflammatory arthritis syndromes or other musculoskeletal disorders. Age-matched controls were selected on the condition that they did not have a hospital diagnosed (ICD-9 or ICD-10) or self-reported musculoskeletal disorders or symptoms.

**deCODE dataset**

The information on hip, knee and vertebral osteoarthritis was obtained from Landspitali University Hospital electronic health records, Akureyri Hospital electronic health records and from a national Icelandic hip or knee arthroplasty registry^1^. Secondary osteoarthritis (e.g. Perthes disease, hip dysplasia), post-trauma osteoarthritis (e.g. ACL rupture) and those also diagnosed with rheumatoid arthritis were excluded from these lists. Only those diagnosed with osteoarthritis after the age of 40 were included. Hand osteoarthritis patients were drawn from a database of 9,000 hand osteoarthritis patients that was initiated in 1972^2^. The study was approved by the Data Protection Authority of Iceland and the National Bioethics Committee of Iceland. Informed consent was obtained from all participants.

**Look-up of known OA loci**

We looked up published OA risk loci in GEFOS, using the index SNP from the corresponding OA GWAS. This look-up did not include OA studies in non-European populations, or loci for cartilage thickness. Of the 18 loci included, 16 were genome-wide significant for OA and two were nominally significant.

**Genetic correlation in paediatric BMD cohort**

We performed LD score regression as described in the main text between all three arcOGEN datasets and a paediatric BMD sample. We used summary data for skull and total body BMD of 9,142 samples from the Avon Longitudinal Study of Parents and their Children (ALSPAC) and the Generation R study. A detailed description of these samples can be found in Ref. 43 in the main text.

**References**

1. Franklin, J., Ingvarsson, T., Englund, M., and Lohmander, S. (2010). Association between occupation and knee and hip replacement due to osteoarthritis: a case-control study. Arthritis Res. Ther. *12*, R102.

2. Styrkarsdottir, U., Thorleifsson, G., Helgadottir, H.T., Bomer, N., Metrustry, S., Bierma-Zeinstra, S., Strijbosch, A.M., Evangelou, E., Hart, D., Beekman, M., et al. (2014). Severe osteoarthritis of the hand associates with common variants within the ALDH1A2 gene and with rare variants at 1p31. Nat. Genet. *46*, 498–502.

**Supplementary Tables**

**Supplementary Tables 3-5.** Significantly associated pathways with FN- or LSBMD at 5% FDR. For each pathway the number of genes, effect estimate (Beta), standard error (SE), p-value from the self-contained analysis (SELF_P), p-value from the competitive analysis (P) and FDR-corrected p-value from the competitive analysis (P_BH) are given. Details on how self-contained and competitive p-values are derived can be found under reference 28 in the main text.

**Supplementary Table 1.** Look-up of OA risk loci in GEFOS. Only studies in European populations were used. P-values and odds ratios (OR) are given for OA from the respective studies, as well as p-values and direction of effect of FN- and LSBMD in GEFOS. EA=effect allele; EAF=effect allele frequency in HapMAp CEU population. *^)^ Loci in linkage disequilibrium.

| **Locus** | **OA SNP** | **MID** | **Comment** | **Site** | **EA** | **OA OR** | **OA P** | **P-value FNBMD** | **Beta_FNBMD (SE)** | **P-value LSBMD** | **Beta_LSBMD (SE)** | **EAF** |
| --- | --- | --- | --- | --- | --- | --- | --- | --- | --- | --- | --- | --- |
| *GDF5* | rs143383 | 20870806 | GWAS | knee | T | 1.16 | 8.30E-09 | 8.44E-02 | -0.002 (0.0081) | 9.99E-04 | -0.005 (0.0081) | 0.67 |
| *DUS4L** | rs4730250 | 21068099 | GWAS | knee | G | 1.17 | 9.17E-09 | 9.85E-01 | -0.001 (0.013) | 9.12E-01 | 0.004 (0.0129) | 0.85 |
| *BCAP29** | rs10953541 | 21068099 | GWAS | knee | T | 1.17 | 3.90E-08 | 4.42E-01 | -0.003 (0.0081) | 7.58E-01 | -0.002 (0.0081) | 0.25 |
| *COG5** | rs3815148 | 20112360 | GWAS | hand and/or knee | C | 1.14 | 8.00E-08 | 3.43E-01 | -0.001 (0.0089) | 9.62E-01 | 0.024 (0.0088) | 0.77 |
| *MCF2L* | rs11842874 | 21871595 | GWAS | knee and/or hip | A | 1.17 | 2.10E-08 | 6.19E-01 | -0.016 (0.0095) | 7.54E-01 | -0.036 (0.0097) | 0.95 |
| *GLT8D1* | rs6976 | 22763110 | GWAS | hip and knee | T | 1.12 | 7.24E-11 | 1.97E-01 | 0.001 (0.0208) | 5.54E-01 | -0.031 (0.0207) | 0.33 |
| *GNL3* | rs11177 | 22763110 | GWAS | hip and knee | A | 1.12 | 1.25E-10 | 1.81E-01 | 0.007 (0.0095) | 5.62E-01 | 0.001 (0.0094) | 0.33 |
| *ASTN2* | rs4836732 | 22763110 | GWAS in females | hip | C | 1.2 | 6.11E-10 | 8.34E-01 | -0.011 (0.0083) | 4.22E-01 | 0.005 (0.0083) | 0.50 |
| *FILIP1/ SENP6* | rs9350591 | 22763110 | GWAS | hip | T | 1.18 | 2.42E-09 | 9.39E-01 | -0.007 (0.0177) | 6.88E-01 | -0.006 (0.0179) | 0.14 |
| *KLHL42/ PTHLH* | rs10492367 | 22763110 | GWAS | hip | T | 1.14 | 1.48E-08 | 2.47E-03 | 0.024 (0.0085) | 9.93E-01 | 0.007 (0.0084) | 0.18 |
| *CHST11* | rs835487 | 22763110 | GWAS | hip | G | 1.13 | 1.64E-08 | 8.96E-01 | -0.032 (0.0107) | 3.95E-01 | -0.005 (0.0106) | 0.68 |
| *TP63* | rs12107036 | 22763110 | females | knee | G | 1.21 | 6.71E-08 | 7.48E-01 | -0.009 (0.0171) | 9.47E-01 | 0.012 (0.0171) | 0.42 |
| *SUPT3H/ CDC5L* | rs10948172 | 22763110 | males | knee and/or hip | G | 1.14 | 7.20E-08 | 7.86E-01 | -0.001 (0.0084) | 2.53E-03 | 0.007 (0.0084) | 0.75 |
| *DOT1L* | rs12982744 | 23505243 | GWAS in males | hip | C | 1.17 | 7.80E-09 | 5.71E-03 | 0.003 (0.0092) | 5.12E-01 | 0.034 (0.0092) | 0.63 |
| *NCOA3* | rs6094710 | 23989986 | GWAS | hip | A | 1.28 | 7.90E-09 | 9.58E-01 | 0.028 (0.0095) | 3.22E-01 | 0.032 (0.0095) | 0.03 |
| *ALDH1A2* | rs4238326 | 24728293 | GWAS | Severe thumbs and severe fingers | C | 1.44 | 8.60E-11 | 8.88E-01 | 0.000 (0.0107) | 4.44E-02 | 0.001 (0.0106) | 0.65 |
| *ALDH1A2* | rs3204689 | 24728293 | GWAS | Severe thumbs and severe fingers | C | 1.46 | 1.10E-11 | 9.46E-01 | 0.001 (0.0085) | 1.89E-02 | -0.021 (0.0085) | 0.45 |
| *CAMK2B* | rs3757837 | 23989986 | suggestive in males | hip | C | 1.27 | 2.20E-06 | 6.88E-01 | -0.011 (0.0083) | 6.24E-01 | 0.005 (0.0083) | 0.92 |
| *IFRD1* | rs5009270 | 23989986 | suggestive | hip | A | 1.1 | 9.00E-07 | 3.68E-03 | 0.009 (0.0097) | 4.43E-03 | 0.004 (0.0096) | 0.23 |

**Supplementary Table 2.** False discovery rate corrected p-values (q-values) for the three genes significantly associated with at least one osteoarthritis (OA) and one bone mineral density (BMD) phenotype. LSBMD=lumbar spine BMD; FNBMD=femoral neck BMD

| **Gene** | **Combined OA** | **Hip OA** | **Knee OA** | **LSBMD** | **FNBMD** |
| --- | --- | --- | --- | --- | --- |
| *COL11A1* | 3.40E-01 | 1.04E-02 | 8.08E-01 | 2.31E-02 | 4.46E-04 |
| *SUPT3H* | 1.40E-01 | 3.74E-02 | 7.55E-01 | 7.27E-04 | 6.98E-01 |
| *APCDD1* | 3.72E-02 | 9.14E-01 | 3.58E-02 | 4.16E-02 | 3.62E-01 |

| **Pathway** | **Genes** | **Beta** | **SE** | **SELF_P** | **P** | **P_BH** |
| --- | --- | --- | --- | --- | --- | --- |
| GO_PLATELET_DERIVED_GROWTH_FACTOR_RECEPTOR_SIGNALING_PATHWAY | 33 | 0.591 | 0.149 | 9.24E-08 | 3.48E-05 | 3.57E-02 |
| GO_MAMMARY_GLAND_EPITHELIUM_DEVELOPMENT | 47 | 0.581 | 0.128 | 2.04E-09 | 2.87E-06 | 5.90E-03 |
| GO_CANONICAL_WNT_SIGNALING_PATHWAY | 81 | 0.408 | 0.101 | 2.36E-12 | 2.64E-05 | 3.26E-02 |
| GO_MAMMARY_GLAND_DEVELOPMENT | 107 | 0.362 | 0.0859 | 1.86E-14 | 1.28E-05 | 1.98E-02 |
| GO_MAMMARY_GLAND_ALVEOLUS_DEVELOPMENT | 14 | 1.73 | 0.274 | 8.67E-15 | 1.34E-10 | 4.12E-07 |
| GO_MAMMARY_GLAND_LOBULE_DEVELOPMENT | 14 | 1.73 | 0.274 | 8.67E-15 | 1.34E-10 | 4.12E-07 |

**Supplementary Table 3.** GO pathways significantly associated with LSBMD when using strict gene annotation.

**Supplementary Table 4.** GO pathways significantly associated with LSBMD when including a 20kb window around genes.

| **Pathway** | **Genes** | **Beta** | **SE** | **SELF_P** | **P** | **P_BH** |
| --- | --- | --- | --- | --- | --- | --- |
| GO_FORMATION_OF_PRIMARY_GERM_LAYER | 107 | 0.30 | 0.08 | 5.85E-09 | 5.58E-05 | 2.06E-02 |
| GO_NEGATIVE_REGULATION_OF_FAT_CELL_DIFFERENTIATION | 41 | 0.64 | 0.15 | 2.76E-05 | 6.41E-06 | 6.39E-03 |
| GO_BRANCH_ELONGATION_OF_AN_EPITHELIUM | 17 | 1.13 | 0.27 | 2.32E-06 | 9.87E-06 | 6.39E-03 |
| GO_MAMMARY_GLAND_EPITHELIUM_DEVELOPMENT | 51 | 0.51 | 0.12 | 8.71E-12 | 1.33E-05 | 7.45E-03 |
| GO_SKELETAL_SYSTEM_DEVELOPMENT | 438 | 0.20 | 0.05 | 1.08E-26 | 8.78E-06 | 6.39E-03 |
| GO_EMBRYO_DEVELOPMENT | 861 | 0.13 | 0.03 | 1.84E-31 | 2.12E-05 | 1.01E-02 |
| GO_CANONICAL_WNT_SIGNALING_PATHWAY | 87 | 0.38 | 0.09 | 2.57E-14 | 9.15E-06 | 6.39E-03 |
| GO_SOMITOGENESIS | 57 | 0.42 | 0.12 | 2.09E-05 | 1.56E-04 | 3.85E-02 |
| GO_PHOSPHATE_CONTAINING_COMPOUND_METABOLIC_PROCESS | 1875 | 0.08 | 0.02 | 4.06E-42 | 8.98E-05 | 2.52E-02 |
| GO_GASTRULATION | 147 | 0.24 | 0.07 | 1.25E-10 | 1.75E-04 | 4.11E-02 |
| GO_MAMMARY_GLAND_DEVELOPMENT | 112 | 0.30 | 0.08 | 3.20E-17 | 8.33E-05 | 2.52E-02 |
| GO_POSITIVE_REGULATION_OF_PEPTIDYL_THREONINE_ PHOSPHORYLATION | 24 | 0.78 | 0.17 | 7.81E-05 | 1.44E-06 | 2.96E-03 |
| GO_EMBRYONIC_MORPHOGENESIS | 524 | 0.14 | 0.04 | 1.99E-21 | 2.00E-04 | 4.11E-02 |
| GO_REGULATION_OF_CATENIN_IMPORT_INTO_NUCLEUS | 26 | 0.58 | 0.16 | 2.77E-04 | 1.91E-04 | 4.11E-02 |
| GO_MAMMARY_GLAND_ALVEOLUS_DEVELOPMENT | 16 | 1.48 | 0.22 | 8.49E-17 | 1.71E-11 | 5.27E-08 |
| GO_REGULATION_OF_PEPTIDYL_THREONINE_PHOSPHORYLATION | 35 | 0.65 | 0.15 | 3.53E-04 | 5.59E-06 | 6.39E-03 |
| GO_MAMMARY_GLAND_LOBULE_DEVELOPMENT | 16 | 1.48 | 0.22 | 8.49E-17 | 1.71E-11 | 5.27E-08 |
| GO_OSTEOBLAST_DEVELOPMENT | 18 | 0.99 | 0.24 | 2.83E-09 | 1.58E-05 | 8.14E-03 |
| GO_DORSAL_VENTRAL_AXIS_SPECIFICATION | 20 | 0.74 | 0.18 | 2.74E-10 | 2.49E-05 | 1.10E-02 |
| GO_MAMMARY_GLAND_EPITHELIAL_CELL_PROLIFERATION | 12 | 1.16 | 0.27 | 5.55E-08 | 1.04E-05 | 6.39E-03 |
| GO_MUSCLE_CELL_DIFFERENTIATION | 230 | 0.22 | 0.06 | 1.64E-15 | 7.21E-05 | 2.34E-02 |
| GO_PHOSPHORYLATION | 1160 | 0.09 | 0.03 | 5.93E-28 | 1.99E-04 | 4.11E-02 |
| GO_SOMITE_DEVELOPMENT | 72 | 0.42 | 0.11 | 1.24E-06 | 3.96E-05 | 1.63E-02 |
| GO_EMBRYONIC_ORGAN_DEVELOPMENT | 391 | 0.21 | 0.05 | 7.25E-21 | 4.84E-06 | 6.39E-03 |
| GO_AXIS_ELONGATION | 26 | 0.69 | 0.18 | 1.01E-05 | 5.69E-05 | 2.06E-02 |
| GO_REGULATION_OF_OSSIFICATION | 170 | 0.25 | 0.07 | 2.04E-23 | 1.85E-04 | 4.11E-02 |
| GO_REGULATION_OF_STEM_CELL_DIFFERENTIATION | 113 | 0.30 | 0.08 | 5.50E-12 | 8.77E-05 | 2.52E-02 |
| GO_BETA_CATENIN_DESTRUCTION_COMPLEX | 14 | 0.79 | 0.22 | 1.95E-04 | 1.22E-04 | 3.13E-02 |
| GO_PROTEIN_COMPLEX_SCAFFOLD | 66 | 0.44 | 0.12 | 1.18E-05 | 6.37E-05 | 2.18E-02 |
| GO_GLUTAMATE_RECEPTOR_BINDING | 35 | 0.55 | 0.16 | 3.98E-08 | 2.16E-04 | 4.30E-02 |
| GO_G_PROTEIN_COUPLED_RECEPTOR_BINDING | 245 | 0.23 | 0.06 | 1.80E-17 | 9.39E-05 | 2.52E-02 |

**Supplementary Table 5.** GO pathways significantly associated with FNBMD when including a 20kb window around genes.

| **Pathway** | **Genes** | **Beta** | **SE** | **SELF_P** | **P** | **P_BH** |
| --- | --- | --- | --- | --- | --- | --- |
| GO_SKELETAL_SYSTEM_DEVELOPMENT | 438 | 0.189 | 0.04 | 5.38E-11 | 8.90E-06 | 2.74E-02 |
| GO_POSITIVE_REGULATION_OF_CARTILAGE_DEVELOPMENT | 28 | 0.657 | 0.16 | 3.81E-09 | 2.14E-05 | 4.41E-02 |
| GO_POSITIVE_REGULATION_OF_CHONDROCYTE_DIFFERENTIATION | 19 | 0.836 | 0.19 | 3.98E-10 | 6.04E-06 | 2.74E-02 |

**Supplementary Table 6.** SNPs taken forward for replication. Summary statistics in the form of effect estimates (BETA), standard errors (SE) and p-values (P) are given for the overall meta-analysis of combined osteoarthritis, as well as each dataset included in it. EA=effect allele; NEA=non-effect allele

|  |  |  |  |  | |  |  | **arcOGEN** | | |  | **UKBB** | | |  | **deCODE** | | |  | **Meta-analysis** | | |
| --- | --- | --- | --- | --- | --- | --- | --- | --- | --- | --- | --- | --- | --- | --- | --- | --- | --- | --- | --- | --- | --- | --- |
| **SNP** | **CHR** | **Closest Gene** | **Gene Distance (bp)** | | **Variant Type** | **EA** | **NEA** | **BETA** | **SE** | **P** |  | **BETA** | **SE** | **P** |  | **BETA** | **SE** | **P** |  | **BETA** | **SE** | **P** |
| rs10133270 | 14 | *TTC7B* | 0 | intron | | G | A | -0.13 | 0.04 | 2.31E-03 |  | -0.08 | 0.04 | 4.95E-02 |  | 0.00 | 0.04 | 9.01E-01 |  | -0.06 | 0.02 | 5.92E-03 |
| rs10170466 | 2 | *AC093901.1* | 112272 | intergenic | | G | T | -0.07 | 0.02 | 2.09E-03 |  | 0.07 | 0.02 | 1.46E-03 |  | 0.01 | 0.02 | 5.93E-01 |  | 0.01 | 0.01 | 5.97E-01 |
| rs10196039 | 2 | *MRPL33* | 23389 | intergenic | | T | C | 0.06 | 0.02 | 4.67E-03 |  | 0.00 | 0.02 | 9.54E-01 |  | -0.01 | 0.02 | 5.00E-01 |  | 0.01 | 0.01 | 2.56E-01 |
| rs10227242 | 7 | *RP11-328J2.1* | 12589 | intergenic | | T | C | -0.15 | 0.06 | 1.21E-02 |  | -0.13 | 0.06 | 1.45E-02 |  | -0.01 | 0.07 | 9.47E-01 |  | -0.11 | 0.04 | 2.39E-03 |
| rs10282661 | 7 | *C7orf10* | 0 | intron | | T | C | 0.03 | 0.02 | 1.25E-01 |  | 0.00 | 0.02 | 8.15E-01 |  | 0.02 | 0.02 | 4.41E-01 |  | 0.02 | 0.01 | 1.45E-01 |
| rs10496575 | 2 | *AC018737.3* | 164676 | intergenic | | A | G | -0.07 | 0.02 | 2.19E-03 |  | 0.01 | 0.02 | 6.26E-01 |  | 0.01 | 0.02 | 7.31E-01 |  | -0.02 | 0.01 | 2.30E-01 |
| rs10512249 | 9 | *PTCH1* | 0 | intron | | A | G | -0.07 | 0.04 | 6.25E-02 |  | -0.05 | 0.03 | 1.78E-01 |  | -0.08 | 0.03 | 1.06E-02 |  | -0.07 | 0.02 | 3.25E-04 |
| rs10518707 | 15 | *SMAD3* | 0 | intron | | A | G | 0.07 | 0.02 | 6.34E-04 |  | 0.04 | 0.02 | 5.97E-02 |  | 0.08 | 0.02 | 7.03E-05 |  | 0.06 | 0.01 | 2.15E-08 |
| rs10808475 | 8 | *TRPS1* | 0 | intron | | C | T | 0.06 | 0.02 | 2.63E-03 |  | 0.03 | 0.02 | 1.03E-01 |  | -0.02 | 0.02 | 3.63E-01 |  | 0.02 | 0.01 | 4.33E-02 |
| rs10852527 | 16 | *HNRNPA1P48* | 121339 | intergenic | | A | G | -0.06 | 0.02 | 9.87E-03 |  | 0.01 | 0.02 | 5.73E-01 |  | -0.01 | 0.02 | 6.10E-01 |  | -0.02 | 0.01 | 1.70E-01 |
| rs10943837 | 6 | *RP11-801I18.1* | 8650 | intergenic | | T | C | 0.04 | 0.02 | 9.01E-02 |  | NA | NA | NA |  | -0.02 | 0.02 | 4.49E-01 |  | 0.01 | 0.02 | 5.78E-01 |
| rs10948155 | 6 | *SUPT3H* | 89097 | intergenic | | C | T | 0.10 | 0.02 | 1.30E-05 |  | 0.04 | 0.02 | 4.84E-02 |  | 0.04 | 0.02 | 6.10E-02 |  | 0.06 | 0.01 | 3.24E-06 |
| rs11012679 | 10 | *CACNB2* | 41205 | intergenic | | C | A | 0.25 | 0.12 | 3.31E-02 |  | 0.02 | 0.10 | 8.78E-01 |  | -0.19 | 0.07 | 4.61E-02 |  | -0.05 | 0.05 | 3.72E-01 |
| rs11049204 | 12 | *RN7SKP15* | 54240 | intergenic | | G | A | 0.12 | 0.03 | 1.59E-05 |  | 0.05 | 0.02 | 4.41E-02 |  | 0.03 | 0.03 | 2.34E-01 |  | 0.06 | 0.01 | 2.15E-05 |
| rs11645645 | 16 | *CLCN7* | 0 | intron | | C | T | -0.02 | 0.02 | 3.23E-01 |  | -0.02 | 0.02 | 2.67E-01 |  | -0.04 | 0.02 | 4.45E-02 |  | -0.03 | 0.01 | 1.27E-02 |
| rs11654856 | 17 | *WSCD1* | 0 | intron | | A | G | -0.05 | 0.03 | 6.31E-02 |  | -0.03 | 0.02 | 2.31E-01 |  | -0.03 | 0.02 | 1.55E-01 |  | -0.04 | 0.01 | 9.44E-03 |
| rs11655490 | 17 | *SLC39A11* | 24713 | intergenic | | A | G | 0.08 | 0.03 | 1.12E-03 |  | 0.00 | 0.02 | 8.49E-01 |  | 0.00 | 0.02 | 8.67E-01 |  | 0.02 | 0.01 | 7.73E-02 |
| rs11659747 | 18 | *RP11-19F9.1* | 457206 | intergenic | | T | C | 0.05 | 0.03 | 1.53E-01 |  | 0.04 | 0.03 | 1.94E-01 |  | 0.02 | 0.03 | 5.03E-01 |  | 0.03 | 0.02 | 5.51E-02 |
| rs11665347 | 18 | *APCDD1* | 0 | intron | | A | G | -0.10 | 0.02 | 8.22E-06 |  | -0.02 | 0.02 | 4.54E-01 |  | -0.03 | 0.02 | 1.21E-01 |  | -0.05 | 0.01 | 1.12E-04 |
| rs11714054 | 3 | *ITPR1* | 0 | intron | | C | T | 0.06 | 0.02 | 1.01E-02 |  | -0.05 | 0.02 | 1.33E-02 |  | 0.01 | 0.02 | 7.10E-01 |  | 0.00 | 0.01 | 8.74E-01 |
| rs11773376 | 7 | *BRAT1* | 0 | intron | | T | C | -0.05 | 0.03 | 9.97E-02 |  | 0.01 | 0.03 | 7.38E-01 |  | -0.02 | 0.03 | 3.71E-01 |  | -0.02 | 0.02 | 2.22E-01 |
| rs11826287 | 11 | *LRP5* | 0 | intron | | C | T | -0.11 | 0.03 | 6.72E-05 |  | 0.00 | 0.02 | 8.52E-01 |  | -0.02 | 0.02 | 4.18E-01 |  | -0.04 | 0.01 | 1.16E-02 |
| rs11881883 | 19 | *FBXO46* | 0 | intron | | A | G | 0.14 | 0.05 | 9.73E-03 |  | -0.01 | 0.05 | 8.29E-01 |  | -0.01 | 0.05 | 8.20E-01 |  | 0.04 | 0.03 | 2.34E-01 |
| rs11964690 | 6 | *SUPT3H* | 0 | intron | | C | T | 0.09 | 0.02 | 4.14E-05 |  | 0.04 | 0.02 | 3.27E-02 |  | 0.07 | 0.02 | 1.21E-03 |  | 0.07 | 0.01 | 1.03E-07 |
| rs11976696 | 7 | *EGFR* | 0 | intron | | G | A | -0.06 | 0.03 | 2.93E-02 |  | 0.01 | 0.02 | 6.01E-01 |  | -0.03 | 0.02 | 2.81E-01 |  | -0.02 | 0.01 | 1.22E-01 |
| rs1201342 | 11 | *C11orf80* | 0 | intron | | G | C | -0.08 | 0.03 | 4.18E-03 |  | -0.06 | 0.02 | 1.47E-02 |  | -0.04 | 0.02 | 9.11E-02 |  | -0.06 | 0.01 | 5.39E-05 |
| rs12086029 | 1 | *RNA5SP45* | 29570 | intergenic | | T | C | 0.06 | 0.02 | 4.57E-03 |  | -0.01 | 0.02 | 6.30E-01 |  | -0.03 | 0.02 | 2.17E-01 |  | 0.01 | 0.01 | 6.62E-01 |
| rs12129980 | 1 | *RP11-76N22.1* | 3987 | downstream gene | | T | C | -0.09 | 0.03 | 1.25E-02 |  | 0.00 | 0.03 | 8.76E-01 |  | -0.17 | 0.17 | 5.15E-01 |  | -0.04 | 0.02 | 1.16E-01 |
| rs12150506 | 17 | *MAPT* | 0 | intron | | A | G | 0.02 | 0.03 | 5.39E-01 |  | NA | NA | NA |  | 0.01 | 0.02 | 8.05E-01 |  | 0.01 | 0.02 | 5.46E-01 |
| rs12190136 | 6 | *SUPT3H* | 9982 | intergenic | | G | A | -0.07 | 0.02 | 1.23E-03 |  | -0.03 | 0.02 | 1.62E-01 |  | -0.03 | 0.02 | 8.73E-02 |  | -0.04 | 0.01 | 3.33E-04 |
| rs12212190 | 6 | *SUPT3H* | 0 | intron | | C | T | 0.10 | 0.02 | 3.90E-05 |  | 0.04 | 0.02 | 4.33E-02 |  | 0.07 | 0.02 | 1.62E-03 |  | 0.07 | 0.01 | 1.98E-07 |
| rs12254219 | 10 | *KCNMA1* | 45167 | intergenic | | T | C | 0.09 | 0.03 | 8.49E-04 |  | 0.02 | 0.03 | 3.93E-01 |  | 0.02 | 0.03 | 3.90E-01 |  | 0.04 | 0.02 | 4.67E-03 |
| rs12474135 | 2 | *AC093166.3* | 61 | upstream gene | | C | T | -0.06 | 0.02 | 5.36E-03 |  | 0.02 | 0.02 | 3.23E-01 |  | -0.04 | 0.02 | 8.29E-02 |  | -0.02 | 0.01 | 4.37E-02 |
| rs1283614 | 5 | *MEF2C-AS1* | 0 | intron | | T | C | 0.12 | 0.04 | 3.77E-03 |  | 0.00 | 0.04 | 9.36E-01 |  | -0.03 | 0.05 | 5.77E-01 |  | 0.04 | 0.03 | 1.45E-01 |
| rs1286063 | 14 | *RPS6KA5* | 0 | intron | | C | T | 0.09 | 0.03 | 8.78E-04 |  | 0.06 | 0.03 | 1.67E-02 |  | 0.00 | 0.04 | 9.56E-01 |  | 0.06 | 0.02 | 3.55E-04 |
| rs1286077 | 14 | *RPS6KA5* | 0 | intron | | C | T | 0.10 | 0.03 | 5.75E-04 |  | 0.06 | 0.03 | 1.74E-02 |  | 0.00 | 0.03 | 9.48E-01 |  | 0.06 | 0.02 | 4.95E-04 |
| rs1286147 | 14 | *RPS6KA5* | 0 | intron | | C | A | 0.09 | 0.03 | 1.23E-03 |  | 0.06 | 0.03 | 2.05E-02 |  | -0.04 | 0.02 | 9.62E-01 |  | 0.04 | 0.01 | 3.77E-03 |
| rs12901071 | 15 | *SMAD3* | 0 | intron | | A | G | 0.08 | 0.02 | 2.92E-04 |  | 0.05 | 0.02 | 2.28E-02 |  | 0.10 | 0.02 | 1.39E-05 |  | 0.08 | 0.01 | 3.12E-10 |
| rs1329710 | 6 | *SUPT3H* | 0 | intron | | T | C | 0.07 | 0.02 | 6.64E-04 |  | 0.02 | 0.02 | 2.20E-01 |  | 0.04 | 0.02 | 2.64E-02 |  | 0.05 | 0.01 | 1.14E-04 |
| rs1524355 | 5 | *RNA5SP189* | 23942 | intergenic | | A | G | -0.08 | 0.02 | 1.69E-03 |  | 0.03 | 0.02 | 1.29E-01 |  | -0.01 | 0.02 | 6.02E-01 |  | -0.02 | 0.01 | 2.63E-01 |
| rs1524928 | 7 | *SHFM1* | 17591 | intergenic | | T | C | -0.05 | 0.02 | 3.69E-02 |  | -0.01 | 0.02 | 5.96E-01 |  | 0.00 | 0.02 | 7.97E-01 |  | -0.01 | 0.01 | 2.06E-01 |
| rs16903255 | 5 | *LINC00461* | 0 | intron | | A | C | 0.12 | 0.05 | 1.90E-02 |  | 0.03 | 0.05 | 5.18E-01 |  | 0.05 | 0.04 | 2.18E-01 |  | 0.06 | 0.03 | 1.87E-02 |
| rs16974630 | 18 | *APCDD1* | 9178 | intergenic | | T | C | -0.09 | 0.02 | 1.03E-04 |  | -0.02 | 0.02 | 3.13E-01 |  | -0.01 | 0.02 | 8.01E-01 |  | -0.03 | 0.01 | 5.56E-03 |
| rs16995654 | 20 | *PLCB4* | 0 | intron | | T | C | -0.13 | 0.05 | 7.07E-03 |  | -0.08 | 0.05 | 1.00E-01 |  | -0.01 | 0.05 | 8.59E-01 |  | -0.07 | 0.03 | 8.92E-03 |
| rs17053584 | 6 | *SGK1* | 0 | intron | | C | G | -0.21 | 0.07 | 3.71E-03 |  | 0.02 | 0.07 | 7.77E-01 |  | 0.02 | 0.06 | 7.12E-01 |  | -0.04 | 0.04 | 2.75E-01 |
| rs17069898 | 18 | *TNFRSF11A* | 0 | intron | | A | G | -0.07 | 0.02 | 1.46E-03 |  | -0.04 | 0.02 | 2.49E-02 |  | 0.00 | 0.04 | 9.81E-01 |  | -0.05 | 0.01 | 3.34E-04 |
| rs17111840 | 14 | *RP11-299L17.3* | 0 | intron | | T | C | 0.10 | 0.05 | 3.54E-02 |  | 0.01 | 0.05 | 7.80E-01 |  | -0.06 | 0.04 | 1.91E-01 |  | 0.01 | 0.03 | 7.17E-01 |
| rs17134820 | 11 | *RP11-111M22.2* | 0 | non-coding transcript | | T | G | -0.07 | 0.05 | 1.58E-01 |  | -0.04 | 0.04 | 2.93E-01 |  | -0.06 | 0.04 | 1.90E-01 |  | -0.06 | 0.03 | 2.43E-02 |
| rs17158899 | 7 | *SEMA3A* | 0 | intron | | T | C | 0.19 | 0.05 | 2.46E-05 |  | 0.02 | 0.04 | 6.13E-01 |  | 0.02 | 0.04 | 6.94E-01 |  | 0.07 | 0.02 | 5.87E-03 |
| rs17264866 | 1 | *PPIEL* | 0 | intron | | G | C | 0.04 | 0.03 | 1.02E-01 |  | 0.03 | 0.02 | 1.53E-01 |  | 0.03 | 0.02 | 1.56E-01 |  | 0.04 | 0.01 | 1.02E-02 |
| rs1734343 | 2 | *PDIA6* | 0 | intron | | G | T | -0.05 | 0.02 | 1.53E-02 |  | 0.01 | 0.02 | 5.96E-01 |  | 0.00 | 0.02 | 9.00E-01 |  | -0.01 | 0.01 | 2.54E-01 |
| rs17412946 | 1 | *KRT8P45* | 7306 | regulatory region | | T | C | 0.08 | 0.03 | 1.28E-02 |  | 0.02 | 0.03 | 5.30E-01 |  | 0.06 | 0.03 | 5.47E-02 |  | 0.05 | 0.02 | 4.77E-03 |
| rs17762165 | 17 | *CRHR1* | 0 | intron | | T | C | 0.01 | 0.03 | 6.16E-01 |  | 0.05 | 0.02 | 3.43E-02 |  | 0.00 | 0.03 | 8.53E-01 |  | 0.02 | 0.01 | 7.91E-02 |
| rs1787663 | 11 | *SCYL1* | 0 | intron | | A | C | -0.06 | 0.02 | 7.77E-03 |  | -0.07 | 0.02 | 8.78E-04 |  | -0.05 | 0.02 | 4.28E-02 |  | -0.06 | 0.01 | 2.42E-06 |
| rs1860710 | 14 | *PAPLN* | 1093 | upstream gene | | T | C | 0.06 | 0.04 | 1.04E-01 |  | 0.00 | 0.03 | 9.28E-01 |  | 0.08 | 0.04 | 2.81E-02 |  | 0.04 | 0.02 | 4.12E-02 |
| rs1903787 | 1 | *COL11A1* | 0 | intron | | G | A | -0.07 | 0.02 | 4.96E-03 |  | -0.05 | 0.02 | 2.45E-02 |  | -0.05 | 0.02 | 4.11E-02 |  | -0.05 | 0.01 | 3.23E-05 |
| rs1957764 | 14 | *STXBP6* | 0 | intron | | G | A | -0.06 | 0.03 | 3.45E-02 |  | 0.02 | 0.03 | 3.98E-01 |  | 0.03 | 0.03 | 2.69E-01 |  | 0.00 | 0.02 | 9.51E-01 |
| rs2052959 | 1 | *WLS* | 0 | intron | | G | T | 0.02 | 0.02 | 4.49E-01 |  | -0.02 | 0.02 | 2.79E-01 |  | -0.02 | 0.02 | 4.46E-01 |  | -0.01 | 0.01 | 4.83E-01 |
| rs2238686 | 19 | *FOSB* | 0 | intron | | C | T | 0.10 | 0.03 | 3.71E-03 |  | 0.00 | 0.03 | 9.86E-01 |  | -0.01 | 0.03 | 6.79E-01 |  | 0.02 | 0.02 | 2.05E-01 |
| rs2246047 | 10 | *RP11-490O24.2* | 19246 | intergenic | | T | G | 0.08 | 0.03 | 1.17E-03 |  | 0.05 | 0.02 | 3.02E-02 |  | -0.01 | 0.02 | 6.93E-01 |  | 0.04 | 0.01 | 5.44E-03 |
| rs2282231 | 1 | *MACF1* | 0 | intron | | T | C | 0.04 | 0.03 | 1.67E-01 |  | 0.04 | 0.02 | 7.06E-02 |  | 0.02 | 0.02 | 3.71E-01 |  | 0.03 | 0.01 | 1.87E-02 |
| rs2395962 | 7 | *NRCAM* | 0 | intron | | G | A | 0.08 | 0.02 | 4.85E-04 |  | -0.03 | 0.02 | 1.38E-01 |  | 0.02 | 0.02 | 2.70E-01 |  | 0.02 | 0.01 | 8.76E-02 |
| rs2398218 | 5 | *CTD-2324F15.2* | 0 | intron | | C | T | 0.10 | 0.04 | 5.70E-03 |  | -0.06 | 0.03 | 8.36E-02 |  | 0.06 | 0.03 | 5.72E-02 |  | 0.03 | 0.02 | 1.17E-01 |
| rs2431613 | 5 | *CTB-181F24.1* | 20280 | intergenic | | G | A | -0.14 | 0.05 | 1.98E-03 |  | 0.02 | 0.04 | 6.44E-01 |  | 0.03 | 0.04 | 4.57E-01 |  | -0.03 | 0.03 | 3.11E-01 |
| rs2457400 | 8 | *RP11-26J3.1* | 0 | non-coding transcript exon | | C | T | -0.04 | 0.02 | 8.09E-02 |  | -0.01 | 0.02 | 7.55E-01 |  | -0.01 | 0.02 | 8.00E-01 |  | -0.02 | 0.01 | 1.93E-01 |
| rs2494454 | 1 | *RP11-416K24.2* | 6908 | intergenic | | C | T | 0.05 | 0.02 | 2.30E-02 |  | 0.04 | 0.02 | 6.40E-02 |  | 0.00 | 0.02 | 9.19E-01 |  | 0.03 | 0.01 | 1.68E-02 |
| rs2513508 | 11 | *C11orf30* | 0 | intron | | C | T | 0.04 | 0.02 | 6.29E-02 |  | -0.03 | 0.02 | 1.01E-01 |  | 0.01 | 0.02 | 7.46E-01 |  | 0.00 | 0.01 | 8.09E-01 |
| rs2669904 | 3 | *ZBTB20* | 0 | intron | | G | A | 0.07 | 0.03 | 1.62E-02 |  | 0.00 | 0.03 | 9.17E-01 |  | -0.01 | 0.02 | 6.33E-01 |  | 0.02 | 0.02 | 3.05E-01 |
| rs2838665 | 21 | *TSPEAR* | 17379 | intergenic | | A | G | -0.06 | 0.02 | 1.16E-02 |  | 0.00 | 0.02 | 9.70E-01 |  | 0.00 | 0.03 | 8.82E-01 |  | -0.02 | 0.01 | 1.33E-01 |
| rs2877561 | 3 | *ILDR1* | 0 | synonymous | | A | C | -0.06 | 0.02 | 1.88E-02 |  | -0.03 | 0.02 | 1.22E-01 |  | 0.03 | 0.02 | 2.21E-01 |  | -0.02 | 0.01 | 1.44E-01 |
| rs3087970 | 15 | *RP11-489D6.2* | 0 | non-coding transcript exon | | G | C | -0.07 | 0.03 | 3.53E-02 |  | -0.03 | 0.03 | 2.64E-01 |  | -0.04 | 0.03 | 2.49E-01 |  | -0.04 | 0.02 | 1.23E-02 |
| rs3116152 | 2 | *DIS3L2* | 0 | intron | | C | G | 0.06 | 0.02 | 4.13E-03 |  | 0.01 | 0.02 | 7.43E-01 |  | 0.03 | 0.02 | 1.04E-01 |  | 0.03 | 0.01 | 5.33E-03 |
| rs3131780 | 1 | *DAB1* | 0 | intron | | G | T | 0.08 | 0.02 | 6.70E-04 |  | 0.03 | 0.02 | 2.17E-01 |  | -0.01 | 0.02 | 6.65E-01 |  | 0.03 | 0.01 | 2.10E-02 |
| rs316405 | 5 | *ZNF131* | 0 | intron | | C | T | 0.07 | 0.02 | 1.57E-03 |  | -0.02 | 0.02 | 2.30E-01 |  | -0.01 | 0.02 | 5.80E-01 |  | 0.01 | 0.01 | 5.19E-01 |
| rs336601 | 3 | *LRRFIP2* | 0 | intron | | A | G | -0.07 | 0.02 | 4.29E-03 |  | 0.01 | 0.02 | 7.66E-01 |  | 0.03 | 0.02 | 2.18E-01 |  | -0.01 | 0.01 | 5.15E-01 |
| rs3755920 | 4 | *CTBP1* | 0 | 5 prime UTR | | C | T | 0.06 | 0.02 | 7.97E-03 |  | -0.01 | 0.02 | 6.85E-01 |  | 0.01 | 0.02 | 7.76E-01 |  | 0.02 | 0.01 | 1.64E-01 |
| rs3755955 | 4 | *IDUA* | 0 | missense | | A | G | -0.02 | 0.03 | 5.89E-01 |  | -0.02 | 0.03 | 3.67E-01 |  | 0.01 | 0.03 | 7.82E-01 |  | -0.01 | 0.02 | 5.04E-01 |
| rs4536164 | 11 | *LGR4* | 0 | intron | | A | C | 0.07 | 0.02 | 1.98E-03 |  | 0.02 | 0.02 | 2.81E-01 |  | 0.03 | 0.02 | 1.44E-01 |  | 0.04 | 0.01 | 1.46E-03 |
| rs465705 | 5 | *CTD-2029E14.1* | 51764 | intergenic | | A | G | -0.09 | 0.04 | 1.02E-02 |  | 0.01 | 0.03 | 6.58E-01 |  | -0.02 | 0.03 | 4.34E-01 |  | -0.03 | 0.02 | 1.13E-01 |
| rs4686872 | 3 | *RTP4* | 25452 | intergenic | | C | T | -0.07 | 0.03 | 1.97E-02 |  | 0.05 | 0.03 | 8.39E-02 |  | 0.01 | 0.03 | 7.59E-01 |  | 0.00 | 0.02 | 9.57E-01 |
| rs4792891 | 17 | *MAPT* | 0 | - | | G | T | -0.03 | 0.02 | 2.61E-01 |  | 0.02 | 0.02 | 2.40E-01 |  | 0.00 | 0.02 | 9.59E-01 |  | 0.00 | 0.01 | 9.11E-01 |
| rs4793022 | 17 | *RP11-209M4.1* | 1051 | upstream gene | | G | C | -0.05 | 0.02 | 2.25E-02 |  | -0.03 | 0.02 | 8.84E-02 |  | 0.01 | 0.02 | 5.43E-01 |  | -0.02 | 0.01 | 5.57E-02 |
| rs4848209 | 2 | *ANAPC1* | 0 | intron | | G | A | 0.07 | 0.02 | 2.88E-03 |  | -0.02 | 0.02 | 3.87E-01 |  | 0.03 | 0.02 | 1.04E-01 |  | 0.03 | 0.01 | 3.50E-02 |
| rs4869742 | 6 | *CCDC170* | 0 | intron | | C | T | 0.00 | 0.02 | 9.86E-01 |  | 0.00 | 0.02 | 9.30E-01 |  | 0.03 | 0.02 | 1.26E-01 |  | 0.01 | 0.01 | 3.06E-01 |
| rs4896622 | 6 | *AIG1* | 0 | intron | | G | A | 0.06 | 0.03 | 1.20E-02 |  | 0.00 | 0.02 | 9.07E-01 |  | 0.03 | 0.02 | 1.81E-01 |  | 0.03 | 0.01 | 2.65E-02 |
| rs4925370 | 20 | *OSBPL2* | 0 | intron | | G | A | 0.06 | 0.02 | 7.54E-03 |  | 0.03 | 0.02 | 1.74E-01 |  | 0.04 | 0.02 | 5.95E-02 |  | 0.04 | 0.01 | 7.37E-04 |
| rs494157 | 1 | *GRIK3* | 13265 | intergenic | | A | G | -0.03 | 0.02 | 1.32E-01 |  | 0.02 | 0.02 | 2.96E-01 |  | 0.00 | 0.02 | 9.21E-01 |  | 0.00 | 0.01 | 8.05E-01 |
| rs4985155 | 16 | *PDXDC1* | 0 | intron | | G | A | 0.02 | 0.02 | 4.58E-01 |  | 0.01 | 0.02 | 5.79E-01 |  | 0.00 | 0.02 | 8.11E-01 |  | 0.01 | 0.01 | 3.81E-01 |
| rs5757762 | 22 | *CACNA1I* | 0 | intron | | A | G | -0.08 | 0.02 | 3.32E-04 |  | 0.01 | 0.02 | 6.91E-01 |  | -0.01 | 0.02 | 6.45E-01 |  | -0.02 | 0.01 | 4.71E-02 |
| rs5766632 | 22 | *UPK3A* | 4940 | downstream gene | | G | A | 0.10 | 0.03 | 4.46E-05 |  | 0.03 | 0.02 | 1.27E-01 |  | 0.08 | 0.02 | 4.71E-04 |  | 0.07 | 0.01 | 6.52E-08 |
| rs5993790 | 22 | *AC000067.1* | 13810 | intergenic | | A | G | -0.10 | 0.03 | 2.57E-03 |  | 0.01 | 0.03 | 8.48E-01 |  | 0.00 | 0.03 | 9.70E-01 |  | -0.02 | 0.02 | 1.63E-01 |
| rs6048946 | 20 | *CST3* | 6227 | intergenic | | T | G | -0.06 | 0.03 | 1.39E-02 |  | 0.01 | 0.02 | 7.35E-01 |  | -0.01 | 0.02 | 7.39E-01 |  | -0.02 | 0.01 | 1.83E-01 |
| rs6060300 | 20 | *EDEM2* | 0 | intron | | C | T | 0.03 | 0.03 | 3.23E-01 |  | -0.03 | 0.03 | 2.65E-01 |  | -0.01 | 0.03 | 7.35E-01 |  | -0.01 | 0.02 | 7.42E-01 |
| rs6094511 | 20 | *EYA2* | 0 | intron | | A | G | 0.03 | 0.03 | 3.42E-01 |  | 0.01 | 0.03 | 6.43E-01 |  | 0.03 | 0.03 | 4.13E-01 |  | 0.02 | 0.02 | 2.01E-01 |
| rs630765 | 6 | *RP1-151F17.2* | 4501 | downstream gene | | G | A | -0.26 | 0.07 | 5.46E-04 |  | -0.08 | 0.07 | 2.43E-01 |  | 0.07 | 0.07 | 3.38E-01 |  | -0.09 | 0.04 | 3.89E-02 |
| rs6826705 | 4 | *RP11-20I20.2* | 0 | intron | | G | A | 0.05 | 0.02 | 2.34E-02 |  | 0.02 | 0.02 | 2.98E-01 |  | -0.01 | 0.02 | 5.19E-01 |  | 0.02 | 0.01 | 1.41E-01 |
| rs6894139 | 5 | *MEF2C-AS1* | 0 | non-coding transcript exon | | T | G | 0.03 | 0.02 | 1.60E-01 |  | 0.02 | 0.02 | 2.72E-01 |  | 0.00 | 0.02 | 9.26E-01 |  | 0.02 | 0.01 | 1.33E-01 |
| rs7022051 | 9 | *WNK2* | 0 | intron | | T | C | -0.08 | 0.03 | 1.49E-02 |  | 0.02 | 0.03 | 5.76E-01 |  | 0.02 | 0.03 | 5.14E-01 |  | -0.01 | 0.02 | 5.60E-01 |
| rs7071206 | 10 | *KCNMA1* | 2963 | upstream gene | | C | T | 0.08 | 0.03 | 3.22E-03 |  | 0.02 | 0.02 | 3.32E-01 |  | 0.03 | 0.03 | 2.82E-01 |  | 0.04 | 0.01 | 4.53E-03 |
| rs7104420 | 11 | *FAT3* | 0 | intron | | G | A | 0.07 | 0.02 | 1.69E-03 |  | -0.01 | 0.02 | 5.47E-01 |  | 0.03 | 0.02 | 1.93E-01 |  | 0.03 | 0.01 | 3.49E-02 |
| rs714031 | 22 | *CACNA1I* | 0 | intron | | T | C | 0.08 | 0.02 | 2.01E-04 |  | -0.01 | 0.02 | 6.07E-01 |  | 0.00 | 0.02 | 8.56E-01 |  | 0.02 | 0.01 | 9.41E-02 |
| rs714784 | 15 | *AP4E1* | 0 | 3 prime UTR | | T | C | -0.06 | 0.02 | 4.88E-03 |  | 0.03 | 0.02 | 1.47E-01 |  | 0.00 | 0.02 | 9.54E-01 |  | -0.01 | 0.01 | 5.06E-01 |
| rs716255 | 10 | *KCNMA1* | 21326 | regulatory region | | T | A | 0.09 | 0.03 | 1.26E-03 |  | 0.03 | 0.03 | 2.78E-01 |  | 0.04 | 0.03 | 1.72E-01 |  | 0.05 | 0.02 | 1.30E-03 |
| rs7174138 | 15 | *GABRG3* | 0 | intron | | G | A | -0.08 | 0.03 | 4.37E-03 |  | 0.02 | 0.03 | 4.90E-01 |  | 0.00 | 0.02 | 9.02E-01 |  | -0.02 | 0.02 | 2.44E-01 |
| rs7217473 | 17 | *CCL15-CCL14* | 0 | intron | | T | C | 0.15 | 0.05 | 1.22E-03 |  | 0.06 | 0.04 | 1.66E-01 |  | 0.00 | 0.05 | 9.17E-01 |  | 0.07 | 0.03 | 6.64E-03 |
| rs7342689 | 16 | *RBBP6* | 17482 | regulatory region | | T | G | 0.09 | 0.03 | 2.74E-03 |  | -0.02 | 0.03 | 4.24E-01 |  | 0.00 | 0.04 | 9.34E-01 |  | 0.02 | 0.02 | 2.31E-01 |
| rs734949 | 10 | *KCNMA1* | 0 | intron | | C | T | 0.06 | 0.02 | 2.83E-03 |  | 0.01 | 0.02 | 4.66E-01 |  | 0.02 | 0.02 | 2.57E-01 |  | 0.03 | 0.01 | 5.99E-03 |
| rs739414 | 16 | *RP11-346C20.3* | 1741 | upstream gene | | C | T | 0.07 | 0.03 | 4.18E-03 |  | 0.04 | 0.02 | 9.34E-02 |  | 0.01 | 0.02 | 5.47E-01 |  | 0.04 | 0.01 | 4.33E-03 |
| rs7545984 | 1 | *RNF220* | 0 | intron | | C | T | 0.22 | 0.06 | 7.22E-04 |  | -0.03 | 0.06 | 6.74E-01 |  | 0.13 | 0.07 | 5.05E-02 |  | 0.10 | 0.04 | 7.44E-03 |
| rs7554123 | 1 | *RNF220* | 0 | intron | | T | G | 0.23 | 0.06 | 3.67E-04 |  | -0.02 | 0.06 | 6.93E-01 |  | 0.13 | 0.07 | 4.15E-02 |  | 0.11 | 0.04 | 4.49E-03 |
| rs7586601 | 2 | *AC074117.10* | 0 | intron | | A | G | -0.06 | 0.02 | 6.99E-03 |  | -0.03 | 0.02 | 1.74E-01 |  | -0.03 | 0.02 | 8.07E-02 |  | -0.04 | 0.01 | 9.80E-04 |
| rs7619689 | 3 | *TRANK1* | 0 | intron | | C | A | 0.07 | 0.02 | 7.92E-04 |  | -0.01 | 0.02 | 7.76E-01 |  | -0.03 | 0.02 | 1.22E-01 |  | 0.01 | 0.01 | 4.52E-01 |
| rs7632108 | 3 | *RP11-259K5.1* | 207 | upstream gene | | A | C | -0.07 | 0.02 | 1.44E-03 |  | 0.01 | 0.02 | 5.77E-01 |  | 0.02 | 0.02 | 2.20E-01 |  | -0.01 | 0.01 | 4.59E-01 |
| rs767919 | 22 | *TTC28* | 0 | intron | | G | A | 0.07 | 0.05 | 1.24E-01 |  | 0.01 | 0.04 | 8.00E-01 |  | -0.02 | 0.05 | 7.43E-01 |  | 0.02 | 0.03 | 3.91E-01 |
| rs7735277 | 5 | *ITGA2* | 0 | intron | | T | C | 0.07 | 0.03 | 2.91E-03 |  | 0.02 | 0.02 | 3.35E-01 |  | 0.03 | 0.02 | 2.12E-01 |  | 0.04 | 0.01 | 3.14E-03 |
| rs7738847 | 6 | *CASC15* | 0 | intron | | T | C | 0.03 | 0.02 | 2.31E-01 |  | 0.00 | 0.02 | 8.93E-01 |  | 0.00 | 0.02 | 9.15E-01 |  | 0.01 | 0.01 | 5.25E-01 |
| rs7788807 | 7 | *FOXK1* | 0 | intron | | C | T | 0.13 | 0.05 | 6.61E-03 |  | -0.04 | 0.04 | 3.36E-01 |  | -0.11 | 0.04 | 1.45E-02 |  | -0.02 | 0.02 | 3.41E-01 |
| rs7894701 | 10 | *DLG5-AS1* | 0 | intron | | G | A | 0.13 | 0.05 | 3.38E-03 |  | -0.15 | 0.04 | 7.32E-04 |  | 0.00 | 0.04 | 9.57E-01 |  | -0.01 | 0.02 | 8.24E-01 |
| rs7956544 | 12 | *RP11-734E19.1* | 10343 | intergenic | | C | T | -0.07 | 0.02 | 2.62E-03 |  | -0.03 | 0.02 | 1.09E-01 |  | 0.00 | 0.03 | 9.74E-01 |  | -0.04 | 0.01 | 4.02E-03 |
| rs7965 | 5 | *MRPL22* | 0 | synonymous | | A | G | 0.11 | 0.04 | 3.77E-03 |  | -0.04 | 0.03 | 2.39E-01 |  | 0.04 | 0.03 | 1.85E-01 |  | 0.03 | 0.02 | 1.38E-01 |
| rs8031440 | 15 | *SMAD3* | 0 | 3 prime UTR | | A | G | -0.07 | 0.03 | 4.01E-03 |  | -0.01 | 0.02 | 5.26E-01 |  | -0.01 | 0.02 | 7.36E-01 |  | -0.03 | 0.01 | 3.25E-02 |
| rs8032675 | 15 | *MAP2K5* | 0 | intron | | T | C | -0.06 | 0.02 | 8.03E-03 |  | -0.02 | 0.02 | 3.69E-01 |  | -0.01 | 0.02 | 6.56E-01 |  | -0.03 | 0.01 | 2.52E-02 |
| rs803798 | 13 | *LINC00348* | 0 | intron | | T | C | -0.07 | 0.03 | 6.66E-03 |  | 0.05 | 0.02 | 1.51E-02 |  | 0.01 | 0.02 | 6.51E-01 |  | 0.00 | 0.01 | 7.82E-01 |
| rs8039089 | 15 | *CYP19A1* | 0 | intron | | G | T | -0.04 | 0.02 | 1.02E-01 |  | 0.02 | 0.02 | 3.23E-01 |  | -0.04 | 0.02 | 3.30E-02 |  | -0.02 | 0.01 | 9.27E-02 |
| rs881803 | 16 | *MYH11* | 0 | intron | | T | C | 0.05 | 0.02 | 4.20E-02 |  | -0.01 | 0.02 | 6.05E-01 |  | 0.00 | 0.02 | 8.36E-01 |  | 0.01 | 0.01 | 5.22E-01 |
| rs884672 | 10 | *RP11-127O4.2* | 33301 | regulatory region | | T | C | -0.08 | 0.04 | 3.66E-02 |  | -0.09 | 0.04 | 2.41E-02 |  | 0.00 | 0.04 | 9.20E-01 |  | -0.05 | 0.02 | 1.57E-02 |
| rs915894 | 6 | *NOTCH4* | 0 | missense | | G | T | 0.03 | 0.02 | 1.26E-01 |  | 0.03 | 0.02 | 2.07E-01 |  | -0.04 | 0.02 | 1.00E-01 |  | 0.01 | 0.01 | 5.79E-01 |
| rs9291326 | 4 | *OCIAD1* | 0 | intron | | A | G | -0.04 | 0.02 | 4.73E-02 |  | -0.01 | 0.02 | 5.85E-01 |  | -0.02 | 0.02 | 2.83E-01 |  | -0.02 | 0.01 | 3.85E-02 |
| rs9318939 | 13 | *RNU6-67P* | 93347 | intergenic | | A | G | 0.06 | 0.04 | 8.04E-02 |  | 0.02 | 0.03 | 5.69E-01 |  | -0.01 | 0.03 | 7.95E-01 |  | 0.02 | 0.02 | 2.90E-01 |
| rs9350354 | 6 | *RP1-135L22.1* | 38118 | regulatory region | | G | A | -0.07 | 0.02 | 1.56E-03 |  | 0.05 | 0.02 | 8.21E-03 |  | -0.04 | 0.02 | 3.45E-02 |  | -0.02 | 0.01 | 1.28E-01 |
| rs9381373 | 6 | *SUPT3H* | 0 | intron | | T | C | 0.07 | 0.02 | 8.29E-04 |  | 0.03 | 0.02 | 1.63E-01 |  | 0.04 | 0.02 | 2.51E-02 |  | 0.05 | 0.01 | 7.56E-05 |
| rs9384514 | 6 | *IYD* | 23927 | intergenic | | G | T | 0.10 | 0.03 | 1.16E-04 |  | 0.04 | 0.02 | 9.89E-02 |  | -0.01 | 0.02 | 7.66E-01 |  | 0.04 | 0.01 | 4.27E-03 |
| rs946328 | 10 | *TLX1* | 0 | intron | | T | C | -0.09 | 0.02 | 4.64E-04 |  | 0.02 | 0.02 | 3.36E-01 |  | 0.07 | 0.03 | 6.59E-03 |  | 0.00 | 0.01 | 9.69E-01 |
| rs9466056 | 6 | *RP1-135L22.1* | 29086 | regulatory region | | A | G | -0.06 | 0.02 | 6.29E-03 |  | 0.03 | 0.02 | 1.73E-01 |  | -0.02 | 0.02 | 2.53E-01 |  | -0.02 | 0.01 | 1.69E-01 |
| rs9526582 | 13 | *ARL11* | 0 | 3 prime UTR | | G | T | -0.04 | 0.02 | 5.81E-02 |  | 0.00 | 0.02 | 7.99E-01 |  | 0.00 | 0.02 | 7.86E-01 |  | -0.01 | 0.01 | 3.28E-01 |
| rs9554402 | 13 | *PSMA6P4* | 82727 | intergenic | | A | T | 0.29 | 0.10 | 3.07E-03 |  | -0.08 | 0.10 | 4.06E-01 |  | -0.05 | 0.07 | 4.78E-01 |  | 0.02 | 0.05 | 6.50E-01 |
| rs9556714 | 13 | *MBNL2* | 7472 | intergenic | | A | G | 0.29 | 0.10 | 2.66E-03 |  | -0.10 | 0.09 | 2.18E-01 |  | -0.05 | 0.07 | 4.72E-01 |  | 0.01 | 0.05 | 8.14E-01 |
| rs9616477 | 22 | *RPL35P8* | 40546 | intergenic | | T | C | 0.06 | 0.03 | 6.75E-02 |  | 0.00 | 0.03 | 9.81E-01 |  | -0.03 | 0.03 | 2.69E-01 |  | 0.00 | 0.02 | 7.94E-01 |
| rs9640740 | 7 | *IFRD1* | 0 | intron | | A | G | 0.05 | 0.02 | 2.25E-02 |  | -0.02 | 0.02 | 3.77E-01 |  | 0.02 | 0.02 | 3.52E-01 |  | 0.02 | 0.01 | 2.08E-01 |
| rs967554 | 1 | *RP5-936J12.1* | 164704 | intergenic | | G | A | -0.05 | 0.03 | 4.25E-02 |  | -0.07 | 0.02 | 2.46E-03 |  | -0.04 | 0.02 | 4.03E-02 |  | -0.05 | 0.01 | 3.07E-05 |
| rs978332 | 13 | *DLEU1* | 0 | intron | | C | T | 0.07 | 0.02 | 1.60E-03 |  | 0.00 | 0.02 | 9.82E-01 |  | 0.02 | 0.02 | 3.63E-01 |  | 0.03 | 0.01 | 2.29E-02 |
| rs9935327 | 16 | *MYH11* | 0 | intron | | A | C | -0.17 | 0.08 | 3.16E-02 |  | 0.05 | 0.08 | 5.48E-01 |  | -0.10 | 0.07 | 2.21E-01 |  | -0.08 | 0.04 | 7.43E-02 |
| rs9945428 | 18 | *FBXO15* | 0 | intron | | C | A | -0.05 | 0.02 | 4.69E-02 |  | 0.02 | 0.02 | 2.82E-01 |  | -0.01 | 0.02 | 7.65E-01 |  | -0.01 | 0.01 | 5.29E-01 |

**Supplementary Table 7.** Results for *SMAD3* in the gene analysis. For each phenotype, false discovery rate corrected (P_BH) and raw p-values (P) as well as the number of SNPs present in the gene are given.

| **Trait** | **SNPs** | **P** | **P_BH** |
| --- | --- | --- | --- |
| Combined OA | 100 | 4.96E-03 | 3.45E-01 |
| Hip OA | 99 | 9.18E-04 | 2.17E-01 |
| Knee OA | 100 | 4.43E-01 | 9.21E-01 |
| FNBMD | 202 | 3.79E-03 | 1.98E-01 |
| LSBMD | 202 | 1.11E-08 | 6.92E-06 |

**Supplementary Table 8.** Sample numbers for all datasets used in this study. Combined, hip and knee refer to hip and/or knee osteoarthritis (OA), hip OA only and knee OA only, respectively. LSBMD=lumbar spine bone mineral density; FNBMD=femoral neck bone mineral density

*arcOGEN excluding all London samples

|  | **arcOGEN** | | | **arcOGEN*** | | | **GEFOS** | | **deCODE** | **UK Biobank** |
| --- | --- | --- | --- | --- | --- | --- | --- | --- | --- | --- |
|  | combined | hip | knee | combined | hip | knee | LSBMD | FNBMD | combined | combined |
| **Cases** | 7,410 | 3,498 | 3,266 | 6,694 | 3,032 | 3,088 | - | - | 9,429 | 6,586 |
| **Controls** | 11,009 | 11,009 | 11,009 | 10,968 | 10,968 | 10,968 | - | - | 199,421 | 26,384 |
| **Total** | 18,419 | 14,507 | 14,275 | 17,662 | 14,000 | 14,056 | 31,800 | 32,961 | 208,850 | 32,970 |

**Supplementary Table 9.** Pathway databases included in the Molecular Signatures Database Canonical Pathways collection.

| **Database** | **URL** |
| --- | --- |
| BioCarta | [http://cgap.nci.nih.gov/Pathways/BioCarta_Pathways](http://www.genecarta.com/) |
| KEGG | <http://www.genome.jp/kegg> |
| Matrisome | [http://matrisomeproject.mit.edu](http://matrisomeproject.mit.edu/) |
| Pathway Interaction Database | [http://pid.nci.nih.gov](http://pid.nci.nih.gov/) |
| Reactome | [http://www.reactome.org](http://www.reactome.org/) |
| SigmaAldrich | <http://www.sigmaaldrich.com/life-science.html> |
| Signaling Gateway | [http://www.signaling-gateway.org](http://www.signaling-gateway.org/) |
| Signal Transduction KE | [http://stke.sciencemag.org](http://stke.sciencemag.org/) |
| SuperArray | [http://www.superarray.com](http://www.superarray.com/) |

**Supplementary Table 10.** In-silico lookup of top SNPs reaching genome-wide significance in any of the CPASSOC analysis for S_het_ or S_hom_, and are not known osteoarthritis (OA) or bone mineral density (BMD) loci, in UK Biobank combined osteoarthritis data. For each SNP summary statistics in the form of effect estimates (BETA), standard errors (SE) and p-values (P). EA=effect allele; NEA=non-effect allele

| **SNP** | **CHR** | **POS** | **EA** | **NEA** | **P** | **BETA** | **SE** |
| --- | --- | --- | --- | --- | --- | --- | --- |
| rs11164649 | 1 | 103444679 | G | T | 3.83E-02 | 0.036 | 0.017 |
| rs996793 | 9 | 23676631 | G | A | 2.55E-01 | -0.061 | 0.054 |
| rs7908390 | 10 | 100147247 | C | T | 3.51E-01 | 0.267 | 0.279 |
| rs7735525 | 5 | 95981203 | G | A | 4.89E-01 | -0.055 | 0.080 |
| rs7853022 | 9 | 18930055 | T | G | 5.33E-01 | 0.014 | 0.022 |
| rs10491510 | 9 | 35040245 | T | C | 5.56E-01 | 0.041 | 0.070 |
| rs12060207 | 1 | 98335381 | T | C | 5.65E-01 | 0.018 | 0.031 |
| rs1748234 | 6 | 45140853 | T | C | 5.91E-01 | -0.009 | 0.016 |
| rs17578878 | 4 | 37900725 | T | C | 6.24E-01 | -0.013 | 0.026 |
| rs2197166 | 18 | 10493908 | A | G | 7.41E-01 | -0.006 | 0.018 |
| rs11188469 | 10 | 97511901 | T | G | 7.42E-01 | 0.010 | 0.031 |
| rs7545984 | 1 | 45003893 | C | T | 7.78E-01 | 0.014 | 0.048 |
| rs17098135 | 14 | 61689992 | C | A | 9.38E-01 | -0.005 | 0.064 |

**Supplementary Table 11.** Genetic correlation (r_g_) as estimated by LD Score Regression between three osteoarthritis (OA) phenotypes and paediatric skull and total body (TB) bone mineral density (BMD), and corresponding p-values (P).

|  | Combined OA | |  | Hip OA | |  | Knee OA | |
| --- | --- | --- | --- | --- | --- | --- | --- | --- |
|  | r_g_ | P |  | r_g_ | P |  | r_g_ | P |
| Skull BMD | 0.1679 | 1.11E-01 |  | 0.0679 | 4.62E-01 |  | 0.1801 | 1.00E-01 |
| TB-BMD | 0.2184 | 1.95E-02 |  | 0.1152 | 2.23E-01 |  | 0.1640 | 1.14E-01 |

**Supplementary Figure 1.** Manhattan plots of multi-trait meta-analysis between osteoarthritis (OA) and lumbar spine bone mineral density (LSBMD). The CPASSOC method was used to calculate the Shom (a-c) and Shet (d-f) statistics for combined OA and LSBMD (a,d), hip OA and LSBMD (b,e) and knee OA and LSBMD (c,f).


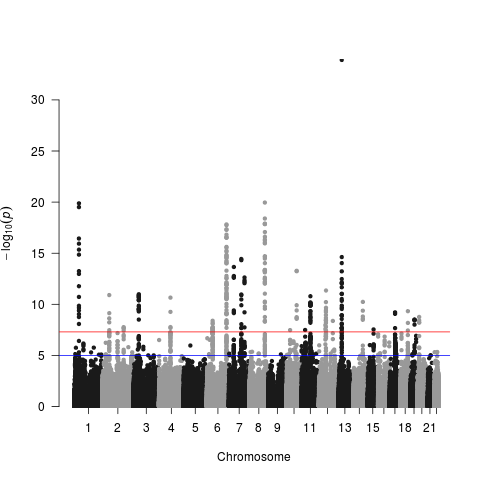

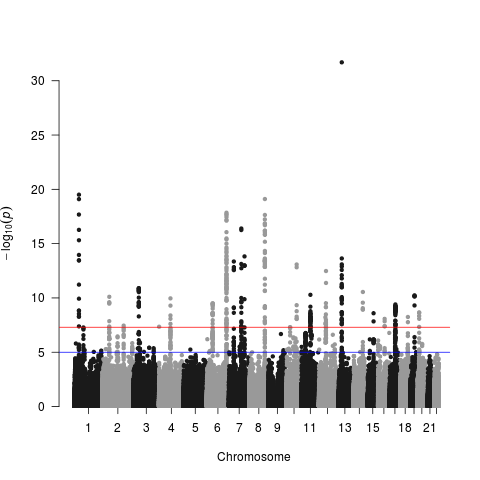

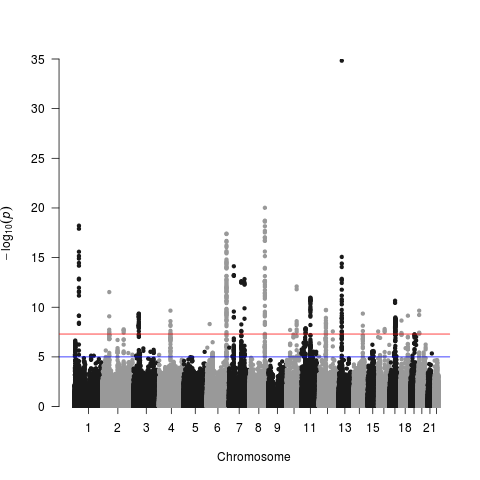

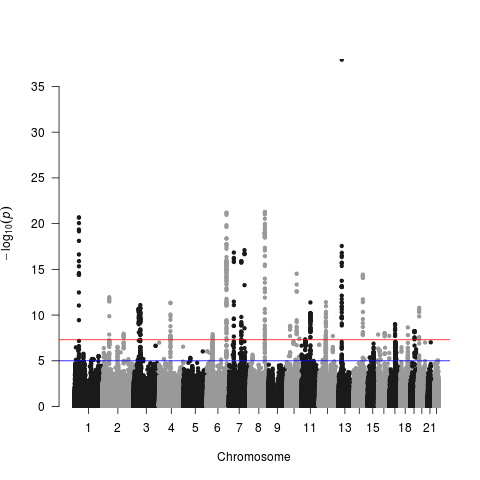

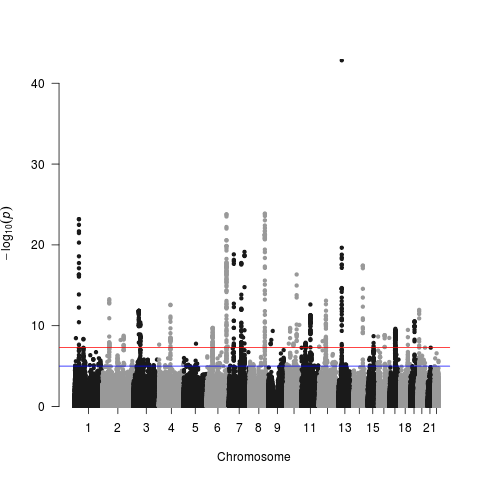

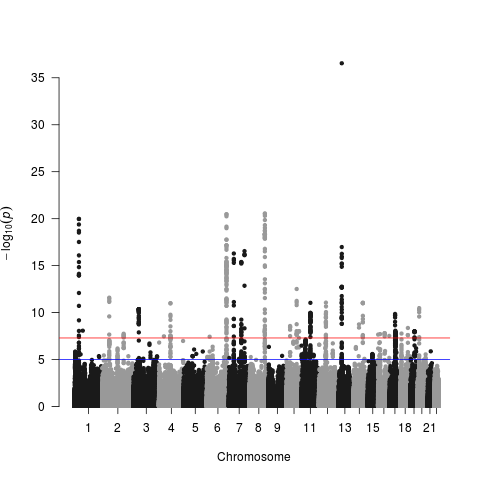


a)

b)

c)

d)

e)

f)

**Supplementary Figure 2.** Manhattan plots of multi-trait meta-analysis between osteoarthritis (OA) and femoral neck bone mineral density (FNBMD). The CPASSOC method was used to calculate the Shom (a-c) and Shet (d-f) statistics for combined OA and FNBMD(a,d), hip OA and FNBMD (b,e) and knee OA and FNBMD (c,f).


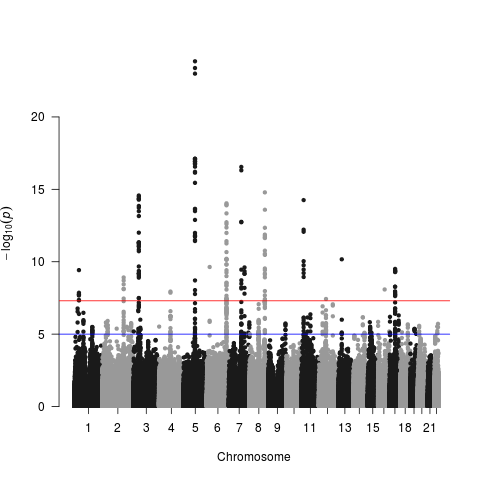

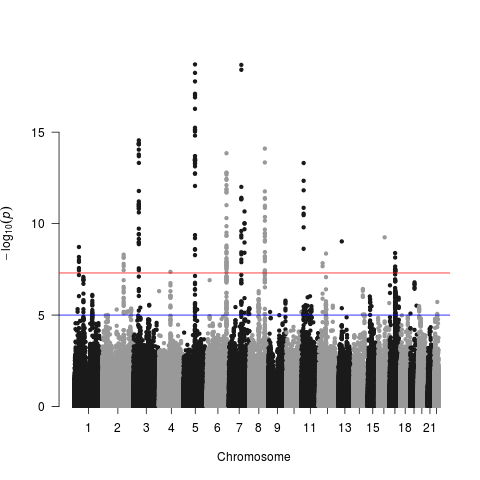

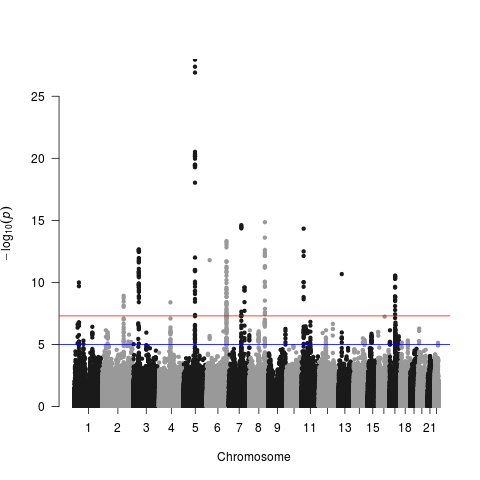

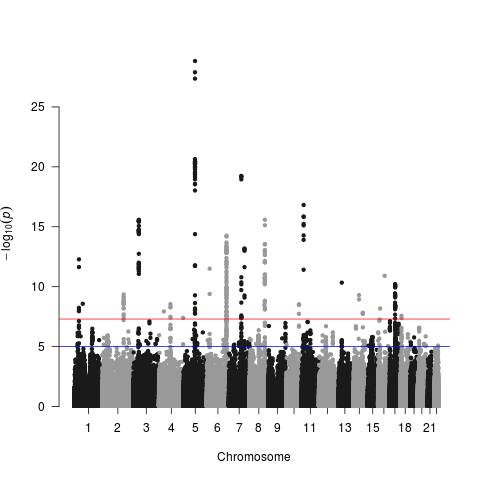

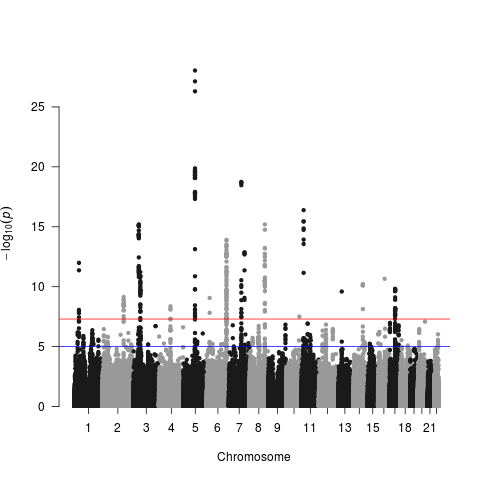

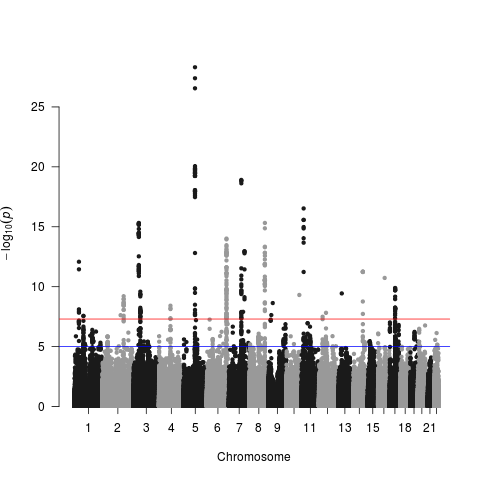


a)

b)

c)

d)

e)

f)

**Supplementary Figure 3.** Quantile-quantile plots of multi-trait meta-analysis between osteoarthritis (OA) and lumbar spine bone mineral density (LSBMD). The CPASSOC method was used to calculate the Shom (a-c) and Shet (d-f) statistics for combined OA and LSBMD(a,d), hip OA and LSBMD (b,e) and knee OA and LSBMD (c,f).


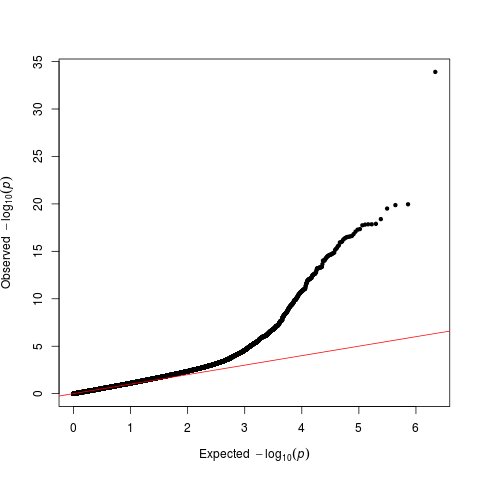

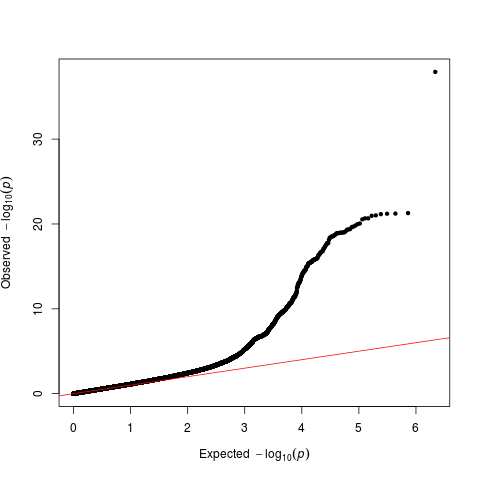

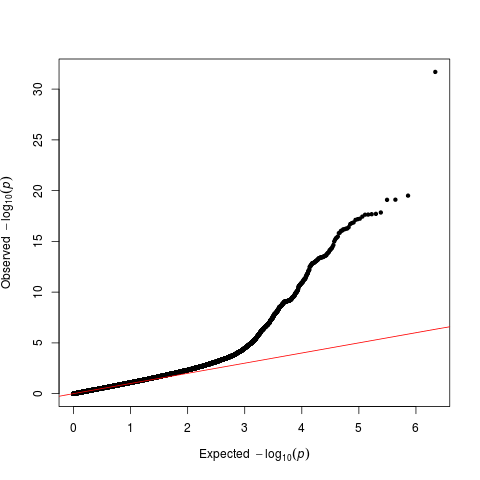

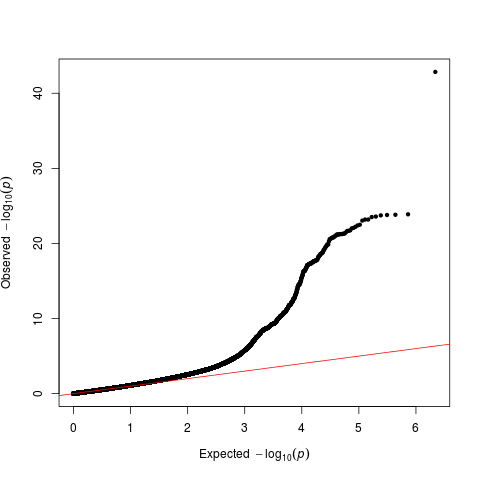

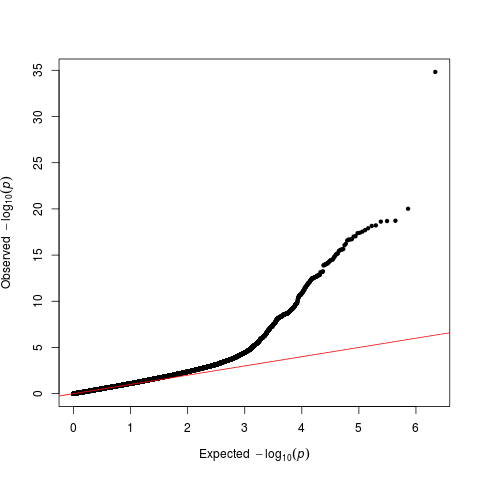

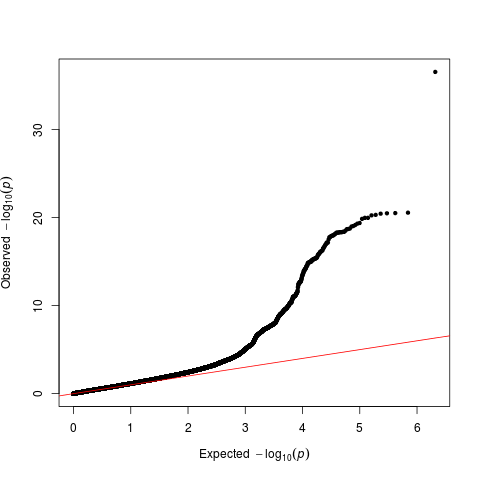


a)

b)

c)

d)

e)

f)

**Supplementary Figure 4.** Quantile-quantile plots of multi-trait meta-analysis between osteoarthritis (OA) and femoral neck bone mineral denisty (FNBMD). The CPASSOC method was used to calculate the Shom (a-c) and Shet (d-f) statistics for combined OA and FNBMD(a,d), hip OA and FNBMD (b,e) and knee OA and FNBMD (c,f).


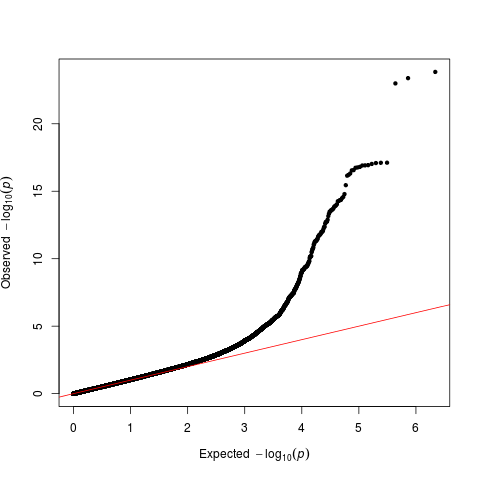

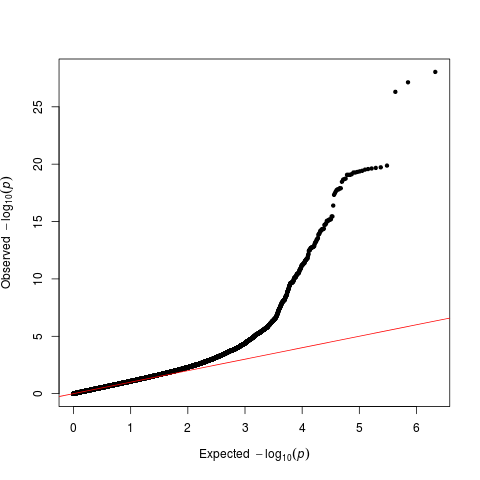

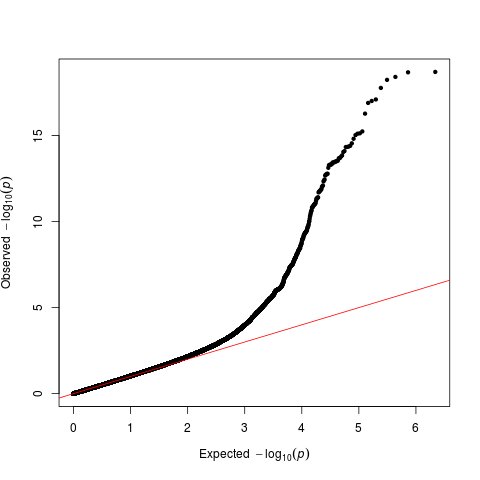

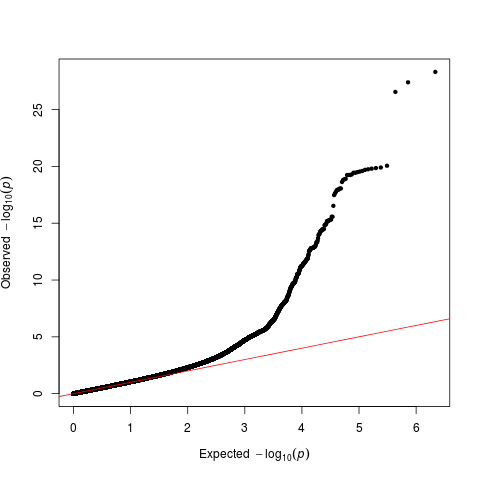

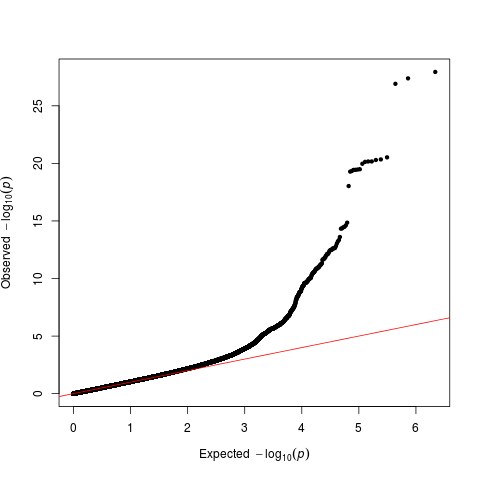

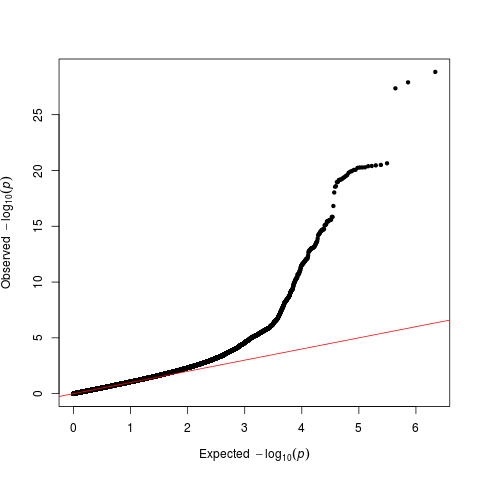


a)

b)

c)

d)

e)

f)

**Supplementary Figure 5.** Mean expression of 15,418 genes in low-grade degenerate articular cartilage of the knee (left) and 16,296 genes in intact articular cartilage of the the hip (right). Mean *SMAD3* expression is shown by a red line. FPKM: fragments per kilobase per million mapped reads. Boxplots represent the median (white dot), interquartile range (IQR; black box) and the lowest and highest value still within 1.5 IQR of the lower and upper quartile, respectively (whiskers).

**
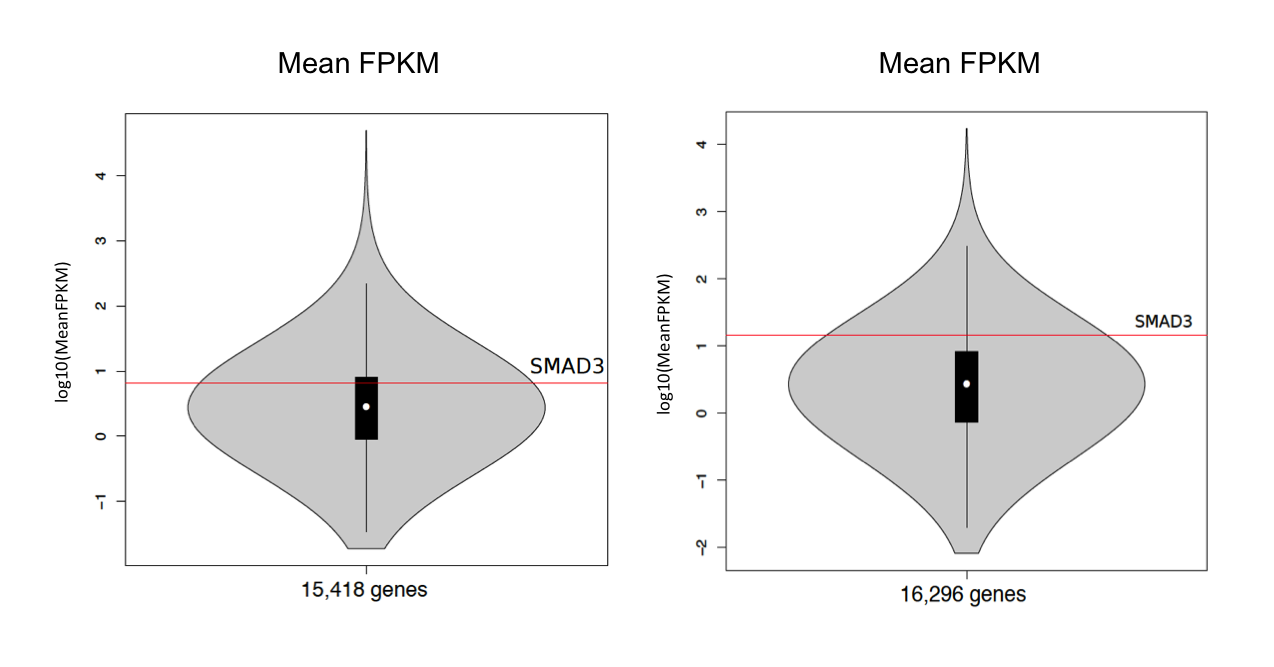
**

**Members of the arcOGEN steering committee**

John Loughlin^1^, Nigel Arden^2^, Fraser Birrell^3,4^, Andrew Carr^2^, Panos Deloukas^5,6^, Michael Doherty^7^, Andrew W. McCaskie^8,9^, William E. R. Ollier^10^, Ashok Rai^11^, Stuart H. Ralston^12^, Tim D. Spector^13^, Ana M. Valdes^7^, Gillian A. Wallis^14^, J. Mark Wilkinson^15^, Eleftheria Zeggini^16^.

Affiliations:

1. Musculoskeletal Research Group, Institute of Cellular Medicine, Newcastle University, Newcastle-upon-Tyne, NE2 4HH, UK.

2. Botnar Research Centre, University of Oxford, Nuffield Orthopaedic Centre, Oxford, OX3 7LD UK.

3. Musculoskeletal Research Group, Institute of Cellular Medicine, Newcastle University, Newcastle upon-Tyne, NE2 4HH, UK.

4. Northumbria Healthcare NHS Foundation Trust, Wansbeck General Hospital, NE63 9JJ, UK.

5. William Harvey Research Institute, Barts and The London School of Medicine and Dentistry, Queen Mary University, London, EC1M 6BQ, UK.

6. Princess Al-Jawhara Al-Brahim Centre of Excellence in Research of Hereditary Disorders (PACER-HD), King Abdulaziz University Jeddah, 21589, Saudi Arabia.

7. Academic Rheumatology, School of Medicine, University of Nottingham, UK, Nottingham, NG5 1PB, UK.

8. Division of Trauma and Orthopaedic Surgery, Department of Surgery, University of Cambridge Cambridge, CB2 0QQ, UK.

9. Musculoskeletal Research Group, Institute of Cellular Medicine, Newcastle University Newcastle-upon-Tyne, NE2 4HH, UK.

10. Centre for Integrated Genomic Medical Research, University of Manchester, Manchester, M13 9PT, UK.

11. Worcestershire Acute Hospitals NHS Trust, Worcester, UK.

12. Centre for Genomic and Experimental Medicine, Institute of Genetics and Molecular Medicine, University of Edinburgh, Edinburgh, EH4 2XU, UK.

13. Department of Twin Research and Genetic Epidemiology, King's College London, London, SE1 7EH, UK.

14. Wellcome Trust Centre for Cell Matrix Research, University of Manchester, Manchester M13 9PT UK.

15. Department of Oncology and Metabolism, University of Sheffield, Sheffield, UK.

16. Wellcome Trust Sanger Institute, Wellcome Genome Campus, Hinxton, CB10 1HH, UK.

**Members of the GEFOS consortium**

Karol Estrada^1,2,3^, Unnur Styrkarsdottir^4,139^, Evangelos Evangelou^5,139^, Yi-Hsiang Hsu^6,7,139^, Emma L Duncan^8,9,139^, Evangelia E Ntzani^5,139^, Ling Oei^1,2,3,139^, Omar M E Albagha^10^, Najaf Amin^2^, John P Kemp^11^, Daniel L Koller^12^, Guo Li^13^, Ching-Ti Liu^14^, Ryan L Minster^15^, Alireza Moayyeri^16,17^, Liesbeth Vandenput^18^, Dana Willner^8,19^, Su-Mei Xiao^20,21^, Laura M Yerges-Armstrong^22^, Hou-Feng Zheng^23^, Nerea Alonso^10^, Joel Eriksson^18^, Candace M Kammerer^15^, Stephen K Kaptoge^16^, Paul J Leo^8^, Gudmar Thorleifsson^4^, Scott G Wilson^17,24,25^, James F Wilson^26,27^, Ville Aalto^28,29^, Markku Alen^30^, Aaron K Aragaki^31^, Thor Aspelund^32,33^, Jacqueline R Center^34,35,36^, Zoe Dailiana^37^, David J Duggan^38^, Melissa Garcia^39^, Natàlia Garcia-Giralt^40^, Sylvie Giroux^41^, Göran Hallmans^42^, Lynne J Hocking^43^, Lise Bjerre Husted^44^, Karen A Jameson^45^, Rita Khusainova^46,47^, Ghi Su Kim^48^, Charles Kooperberg^31^, Theodora Koromila^49^, Marcin Kruk^50^, Marika Laaksonen^51^, Andrea Z Lacroix^31^, Seung Hun Lee^48^, Ping C Leung^52^, Joshua R Lewis^24,25^, Laura Masi^53^, Simona Mencej-Bedrac^54^, Tuan V Nguyen^34,35^, Xavier Nogues^40^, Millan S Patel^55^, Janez Prezelj^56^, Lynda M Rose^57^, Serena Scollen^58^, Kristin Siggeirsdottir^32^, Albert V Smith^32,33^, Olle Svensson^59^, Stella Trompet^60,61^, Olivia Trummer^62^, Natasja M van Schoor^63^, Jean Woo^64^, Kun Zhu^24,25^, Susana Balcells^65^, Maria Luisa Brandi^53^, Brendan M Buckley^66^, Sulin Cheng^67,68^, Claus Christiansen^69^, Cyrus Cooper^45^, George Dedoussis^70^, Ian Ford^71^, Morten Frost^72,73^, David Goltzman^74^, Jesús González-Macías^75,76^, Mika Kähönen^77,78^, Magnus Karlsson^79^, Elza Khusnutdinova^46,47^, Jung-Min Koh^48^, Panagoula Kollia^49^, Bente Lomholt Langdahl^44^, William D Leslie^80^, Paul Lips^81,82^, Östen Ljunggren^83^, Roman S Lorenc^50^, Janja Marc^54^, Dan Mellström^18^, Barbara Obermayer-Pietsch^62^, José M Olmos^75,76^, Ulrika Pettersson-Kymmer^84^, David M Reid^43^, José A Riancho^75,76^, Paul M Ridker^57,85^, François Rousseau^41,86,87^, P Eline Slagboom^88,3^, Nelson LS Tang^89,90^, Roser Urreizti^65^, Wim Van Hul^91^, Jorma Viikari^92,93^, María T Zarrabeitia^94^, Yurii S Aulchenko^2^, Martha Castano-Betancourt^1,2,3^, Elin Grundberg^95,96,97^, Lizbeth Herrera^1^, Thorvaldur Ingvarsson^98,99,33^, Hrefna Johannsdottir^4^, Tony Kwan^95,96^, Rui Li^100^, Robert Luben^16^, Carolina Medina-Gómez^1,2^, Stefan Th Palsson^4^, Sjur Reppe^101^, Jerome I Rotter^102^, Gunnar Sigurdsson^103,33^, Joyce B J van Meurs^1,2,3^, Dominique Verlaan^95,96^, Frances MK Williams^17^, Andrew R Wood^104^, Yanhua Zhou^14^, Kaare M Gautvik^101,105,106^, Tomi Pastinen^95,96,107^, Soumya Raychaudhuri^108,109^, Jane A Cauley^110^, Daniel I Chasman^57,85^, Graeme R Clark^8^, Steven R Cummings^111^, Patrick Danoy^8^, Elaine M Dennison^45^, Richard Eastell^112^, John A Eisman^34,35,36^, Vilmundur Gudnason^32,33^, Albert Hofman^2,3^, Rebecca D Jackson^113,114^, Graeme Jones^115^, J Wouter Jukema^60,116,117^, Kay-Tee Khaw^16^, Terho Lehtimäki^118,119^, Yongmei Liu^120^, Mattias Lorentzon^18^, Eugene McCloskey^112,121^, Braxton D Mitchell^22^, Kannabiran Nandakumar^6,7^, Geoffrey C Nicholson^122^, Ben A Oostra^123^, Munro Peacock^124^, Huibert A P Pols^1,2^, Richard L Prince^24,25^, Olli Raitakari^28,29^, Ian R Reid^125^, John Robbins^126^, Philip N Sambrook^127^, Pak Chung Sham^128,129^, Alan R Shuldiner ^22,130^, Frances A Tylavsky^131^, Cornelia M van Duijn^2^, Nick J Wareham^132^, L Adrienne Cupples^14,133^, Michael J Econs^124,12^, David M Evans^11^, Tamara B Harris^39^, Annie Wai Chee Kung^20,21^, Bruce M Psaty^134,135^, Jonathan Reeve^136^, Timothy D Spector^17^, Elizabeth A Streeten^22,130^, M Carola Zillikens^1^, Unnur Thorsteinsdottir^4,33,140^, Claes Ohlsson^18,140^, David Karasik^6,7,140^, J Brent Richards^137,17,140^, Matthew A Brown^8,140^, Kari Stefansson^4,33,140^, André G Uitterlinden^1,2,3,140^, Stuart H Ralston^10,140^, John P A Ioannidis^138,5,140^, Douglas P Kiel^6,7,140^, Fernando Rivadeneira^1,2,3,140^

Affiliations:

^1^Department of Internal Medicine, Erasmus Medical Center, Rotterdam, The Netherlands.  ^2^Department of Epidemiology, Erasmus Medical Center, Rotterdam, The Netherlands.  ^3^Netherlands Genomics Initiative (NGI)-sponsored Netherlands Consortium for Healthy Aging (NCHA), Leiden, The Netherlands.  ^4^deCODE Genetics, Reykjavik, Iceland.  ^5^Department of Hygiene and Epidemiology, University of Ioannina, Ioannina, Greece.  ^6^Institute for Aging Research, Hebrew SeniorLife, Boston, USA.  ^7^Department of Medicine, Harvard Medical School, Boston, USA.  ^8^Human Genetics Group, University of Queensland Diamantina Institute, Brisbane, Australia.  ^9^Department of Endocrinology, Royal Brisbane and Women's Hospital, Brisbane, Australia.  ^10^Rheumatic Diseases Unit, Institute of Genetics and Molecular Medicine, University of Edinburgh, Edinburgh, UK.  ^11^Medical Research Council (MRC) Centre for Causal Analyses in Translational Epidemiology, University of Bristol, Bristol, UK.  ^12^Department of Medical and Molecular Genetics, Indiana University School of Medicine, Indianapolis, USA.  ^13^Cardiovascular Health Research Unit, University of Washington, Seattle, USA.  ^14^Department of Biostatistics, Boston University School of Public Health, Boston, USA.  ^15^Department of Human Genetics, University of Pittsburgh, Pittsburgh, PA, USA.  ^16^Department of Public Health and Primary Care, University of Cambridge, Cambridge, UK.  ^17^Department of Twin Research and Genetic Epidemiology, King's College London, London, UK.  ^18^Centre for Bone and Arthritis Research, Institute of Medicine, Sahlgrenska Academy, University of Gothenburg, Gothenburg, Sweden.  ^19^Australian Centre for Ecogenomics, University of Queensland, Brisbane, Australia.  ^20^Department of Medicine, The University of Hong Kong, Hong Kong, China.  ^21^Research Centre of Heart, Brain, Hormone and Healthy Aging, The University of Hong Kong, Hong Kong, China.  ^22^Department of Medicine, Division of Endocrinology, Diabetes and Nutrition, University of Maryland School of Medicine, Baltimore, MD, USA.  ^23^Department of Human Genetics, Lady Davis Institute, McGill University, Montreal, Canada.  ^24^School of Medicine and Pharmacology, University of Western Australia, Perth, Australia.  ^25^Department of Endocrinology and Diabetes, Sir Charles Gairdner Hospital, Perth, Australia.  ^26^Centre for Population Health Sciences, University of Edinburgh, Edinburgh, UK.  ^27^MRC Human Genetics Unit, MRC Institute of Genetics and Molecular Medicine at the University of Edinburgh, Edinburgh, UK.  ^28^Department of Clinical Physiology, Turku University Hospital, Turku, Finland.  ^29^Research Centre of Applied and Preventive Cardiovascular Medicine, University of Turku, Turku, Finland.  ^30^Department of Medical Rehabilitation, Oulu University Hospital and Institute of Health Sciences, Oulu, Finland.  ^31^Division of Public Health Sciences, Fred Hutchinson Cancer Research Center, Seattle, USA.  ^32^Icelandic Heart Association, Kopavogur, Iceland.  ^33^Faculty of Medicine, University of Iceland, Reykjavik, Iceland.  ^34^Osteoporosis and Bone Biology Program, Garvan Institute of Medical Research, Sydney, Australia.  ^35^Department of Medicine, University of New South Wales, Sydney, Australia.  ^36^Department of Endocrinology, St Vincents Hospital, Sydney, Australia.  ^37^Department of Orthopaedic Surgery, Medical School University of Thessalia, Larissa, Greece.  ^38^Translational Genomics Research Institute, Phoenix, USA.  ^39^Laboratory of Epidemiology, Demography, and Biometry, National Institute on Aging, Bethesda, MD, USA.  ^40^Department of Internal Medicine, Hospital del Mar, Instituto Municipal de Investigación Médica (IMIM), Red Temática de Investigación Cooperativa en Envejecimiento y Fragilidad (RETICEF), Universitat Autònoma de Barcelona (UAB), Barcelone, Spain. ^41^Unité de recherche en génétique humaine et moléculaire, Centre de recherche du Centre hospitalier universitaire de Québec - Hôpital St-François-d'Assise (CHUQ/HSFA), Québec City, Canada.  ^42^Department of Public Health and Clinical Medicine, Umeå Unviersity, Umeå, Sweden.  ^43^Musculoskeletal Research Programme, Division of Applied Medicine, University of Aberdeen, Aberdeen, UK.  ^44^Department of Endocrinology and Internal Medicine, Aarhus University Hospital, Aarhus C, Denmark.  ^45^MRC Lifecourse Epidemiology Unit, University of Southampton, Southampton, UK.  ^46^Ufa Scientific Centre of Russian Academy of Sciences, Institute of Biochemistry and Genetics, Ufa, Russia.  ^47^Biological Department, Bashkir State University, Ufa, Russia.  ^48^Division of Endocrinology and Metabolism, Asan Medical Center, University of Ulsan College of Medicine, Seoul, South Korea.  ^49^Department of Genetics and Biotechnology, Faculty of Biology, University of Athens, Athens, Greece.  ^50^Department of Biochemistry and Experimental Medicine, The Children's Memorial Health Institute, Warsaw, Poland.  ^51^Department of Food and Environmental Sciences, University of Helsinki, Helsinki, Finland.  ^52^Jockey Club Centre for Osteoporosis Care and Control, The Chinese University of Hong Kong, Hong Kong SAR, China.  ^53^Department of Internal Medicine, University of Florence, Florence, Italy.  ^54^Department of Clinical Biochemistry, University of Ljubljana, Ljubljana, Slovenia.  ^55^Department of Medical Genetics, University of British Columbia, Vancouver, Canada.  ^56^Department of Endocrinology, University Medical Center, Ljubljana, Slovenia.  ^57^Division of Preventive Medicine, Brigham and Women's Hospital, Boston, USA.  ^58^Department of Medicine, University of Cambridge, Cambridge, UK.  ^59^Department of Surgical and Perioperative Sciences, Umeå Unviersity, Umeå, Sweden.  ^60^Department of Cardiology, Leiden University Medical Center, Leiden, The Netherlands.  ^61^Department of Gerontology and Geriatrics, Leiden University Medical Center, Leiden, The Netherlands.  ^62^Department of Internal Medicine, Division of Endocrinology and Metabolism, Medical University Graz, Graz, Austria.  ^63^Department of Epidemiology and Biostatistics, Extramuraal Geneeskundig Onderzoek (EMGO) Institute for Health and Care Research, Vrije Universiteit (VU) University Medical Center, Amsterdam, The Netherlands.  ^64^Department of Medicine and Therapeutics, The Chinese University of Hong Kong, Hong Kong SAR, China.  ^65^Department of Genetics, University of Barcelona, Centro de Investigación Biomédica en Red de Enfermedades Raras (CIBERER), Institut de Biomedicina de la Universitat de Barcelona (IBUB), Barcelone, Spain.  ^66^Department of Pharmacology and Therapeutics, University College Cork, Cork, Ireland.  ^67^Department of Health Sciences, University of Jyväskylä, Jyväskylä, Finland.  ^68^Department of Orthopaedics and Traumatology, Kuopio University Hospital, Kuopio, Finland.  ^69^Center for Clinical and Basic Research (CCBR)-Synarc, Ballerup, Denmark.  ^70^Department of Nutrition and Dietetics, Harokopio University, Athens, Greece.  ^71^Robertson Center for Biostatistics, University of Glasgow, Glasgow, United Kingdom.  ^72^Department of Endocrinology, Odense University Hospital, Odense, Denmark.  ^73^Clinical Institute, University of Southern Denmark, Odense, Denmark.  ^74^Department of Medicine, McGill University, Montreal, Canada.  ^75^Department of Medicine, University of Cantabria, Santander, Spain.  ^76^Department of Internal Medicine, Hospital Universitario Marqués de Valdecilla and Instituto de Formación e Investigación Marqués de Valdecilla (IFIMAV), Santander, Spain.  ^77^Department of Clinical Physiology, Tampere University Hospital, Tampere, Finland.  ^78^Department of Clinical Physiology, University of Tampere School of Medicine, Tampere, Finland.  ^79^Clinical and Molecular Osteoporosis Research Unit, Department of Clinical Sciences and Department of Orthopaedics, Lund University, Malmö, Sweden.  ^80^Department of Internal Medicine, University of Manitoba, Winnipeg, Canada.  ^81^Department of Endocrinology, Vrije Universiteit (VU) University Medical Center, Amsterdam, The Netherlands.  ^82^Extramuraal Geneeskundig Onderzoek (EMGO) Institute for Health and Care Research, Vrije Universiteit (VU) University Medical Center, Amsterdam, The Netherlands.  ^83^Department of Medical Sciences, University of Uppsala, Uppsala, Sweden.  ^84^Department of Pharmacology and Neuroscience, Umeå University, Umeå, Sweden.  ^85^Harvard Medical School, Boston, USA.  ^86^Department of Molecular Biology, Medical Biochemistry and Pathology, Université Laval, Québec City, Canada.  ^87^The APOGEE-Net/CanGèneTest Network on Genetic Health Services and Policy, Université Laval, Québec City, Canada.  ^88^Department of Molecular Epidemiology, Leiden University Medical Center, Leiden, The Netherlands.  ^89^Department of Chemical Pathology, The Chinese University of Hong Kong, Hong Kong SAR, China.  ^90^Li Ka Shing Institute of Health Sciences, The Chinese University of Hong Kong, Hong Kong SAR, China.  ^91^Department of Medical Genetics, University of Antwerp, Antwerp, Belgium.  ^92^Department of Medicine, Turku University Hospital, Turku, Finland.  ^93^Department of Medicine, University of Turku, Turku, Finland.  ^94^Department of Legal Medicine, University of Cantabria, Santander, Spain.  ^95^Department of Human Genetics, McGill University, Montreal, Canada.  ^96^McGill University and Genome Québec Innovation Centre, Montreal, Canada.  ^97^Wellcome Trust Sanger Institute, Hinxton, UK.  ^98^Department of Orthopedic Surgery, Akureyri Hospital, Akureyri, Iceland.  ^99^Institution of Health Science, University Of Akureyri, Akureyri, Iceland.  ^100^Department of Epidemiology and Biostatistics, Lady Davis Institute, McGill University, Montreal, Canada.  ^101^Department of Medical Biochemistry, Oslo University Hospital, Oslo, Norway.  ^102^Medical Genetics Institute, Cedars-Sinai Medical Center, Los Angeles, USA.  ^103^Department of Endocrinology and Metabolism, University Hospital, Reykjavik, Iceland.  ^104^Genetics of Complex Traits, Peninsula College of Medicine and Dentistry, University of Exeter, Exeter, England.  ^105^Department of Clinical Biochemistry, Lovisenberg Deacon Hospital, Oslo, Norway.  ^106^Institute of Basic Medical Sciences, University of Oslo, Oslo, Norway.  ^107^Department of Medical Genetics, McGill University Health Centre, Montreal, Canada.  ^108^Division of Genetics and Rheumatology, Brigham and Women's Hospital, Harvard Medical School, Boston, United States.  ^109^Program in Medical and Population Genetics, Broad Institute, Cambridge, United States.  ^110^Department of Epidemiology, University of Pittsburgh, Pittsburgh, USA.  ^111^California Pacific Medical Center, San Francisco, CA, USA.  ^112^National Institute for Health and Research (NIHR) Musculoskeletal Biomedical Research Unit, University of Sheffield, Sheffield, UK.  ^113^Department of Internal Medicine, The Ohio State University, Columbus, USA.  ^114^Center for Clinical and Translational Science, The Ohio State University, Columbus, USA.  ^115^Menzies Research Institute, University of Tasmania, Hobart, Australia.  ^116^Durrer Center for Cardiogenetic Research, Amsterdam, The Netherlands.  ^117^Interuniversity Cardiology Institute of the Netherlands, Utrecht, The Netherlands.  ^118^Department of Clinical Chemistry, Tampere University Hospital, Tampere, Finland.  ^119^Department of Clinical Chemistry, University of Tampere School of Medicine, Tampere, Finland.  ^120^Center for Human Genomics, Wake Forest University School of Medicine, Winston-Salem, NC, USA.  ^121^Academic Unit of Bone Metabolism, Metabolic Bone Centre, University of Sheffield, Sheffield, UK.  ^122^Rural Clinical School, The University of Queensland, Toowoomba, Australia.  ^123^Department of Clinical Genetics, Erasmus Medical Center, Rotterdam, The Netherlands.  ^124^Department of Medicine, Indiana University School of Medicine, Indianapolis, USA.  ^125^Department of Medicine, University of Auckland, Auckland, New Zealand.  ^126^Department of Medicine, University of Davis, Sacramento, CA, USA.  ^127^Kolling Institute, Royal North Shore Hospital, University of Sydney, Sydney, Australia.  ^128^Department of Psychiatry, The University of Hong Kong, Hong Kong, China.  ^129^Centre for Reproduction, Development and Growth, The University of Hong Kong, Hong Kong, China.  ^130^Geriatric Research and Education Clinical Center (GRECC), Veterans Administration Medical Center, Baltimore, MD, USA.  ^131^Department of Preventive Medicine, University of Tennessee College of Medicine, Memphis, TN, USA.  ^132^MRC Epidemiology Unit Box 285, Medical Research Council, Cambridge, UK.  ^133^Framingham Heart Study, Framingham, USA.  ^134^Departments of Medicine, Epidemiology and Health Services, University of Washington, Seattle, USA.  ^135^Group Health Research Institute, Group Health Cooperative, Seattle, USA.  ^136^Medicine box 157, University of Cambridge, Cambridge, UK.  ^137^Departments of Medicine, Human Genetics, Epidemiology and Biostatistics, Lady Davis Institute, McGill University, Montreal, Canada.  ^138^Stanford Prevention Research Center, Stanford University, Stanford, USA.
